# Gini's mean difference and the long-term prognostic value of nodal quanta classes after pre-operative chemotherapy in advanced breast cancer

**DOI:** 10.1038/s41598-022-07078-7

**Published:** 2022-02-22

**Authors:** Vincent Vinh-Hung, Hilde Van Parijs, Olena Gorobets, Christel Fontaine, Nam P. Nguyen, Bhumsuk Keam, Dung Minh Nguyen, Mark De Ridder

**Affiliations:** 1 Oncologisch Centrum, Universitair Ziekenhuis (UZ) Brussel, Vrije Universiteit Brussel, Laarbeeklaan 101, 1090 Brussels, Belgium; 2 University Hospital of Martinique, Fort-de-France, Martinique France; 3 Centre Hospitalier de La Polynésie Française, Papeete, French Polynesia France; 4 Ukrainian Military Medical Academy, Moskovska Street, Kyiv, Ukraine; 5 Radiation Oncology, Howard University, Washington, DC USA; 6 Department of Internal Medicine, Seoul National University Hospital, Seoul, Republic of Korea; 7 Hospital of Orthopedics and Rehabilitation, Ho Chi Minh City, Vietnam

**Keywords:** Breast cancer, Applied mathematics, Surgical oncology, Prognostic markers

## Abstract

Gini's mean difference (GMD, mean absolute difference between any two distinct quantities) of the restricted mean survival times (RMSTs, expectation of life at a given time limit) has been proposed as a new metric where higher GMD indicates better prognostic value. GMD is applied to the RMSTs at 25 years time-horizon to evaluate the long-term overall survival of women with breast cancer who received neoadjuvant chemotherapy, comparing a classification based on the number (pN) versus a classification based on the ratio (LNRc) of positive nodes found at axillary surgery. A total of 233 patients treated in 1980–2009 with documented number of positive nodes (*npos*) and number of nodes examined (*ntot*) were identified. The numbers were categorized into pN0, *npos* = 0; pN1, *npos* = [1,3]; pN2, *npos* = [4,9]; pN3, *npos* ≥ 10. The ratios *npnx* = *npos*/*ntot* were categorized into Lnr0, *npnx* = 0; Lnr1, *npnx* = (0,0.20]; Lnr2, *npnx* = (0.20,0.65]; Lnr3, *npnx* > 0.65. The GMD for pN-classification was 5.5 (standard error: ± 0.9) years, not much improved over a simple node-negative vs. node-positive that showed a GMD of 5.0 (± 1.4) years. The GMD for LNRc-classification was larger, 6.7 (± 0.8) years. Among other conventional metrics, Cox-model LNRc's c-index was 0.668 vs. pN's c = 0.641, indicating commensurate superiority of LNRc-classification. The usability of GMD-RMSTs warrants further investigation.

## Introduction

The evaluation of a prognostic marker entails three inseparable facets: first, what is the nature of the marker; second, what are and how to measure the prognostic outcomes of interest; and third, how to compare the outcomes.

On the first facet, we pioneered various nodal quanta in breast cancer^1–3^ quanta short for quantitative, borrowed from the Covid literature^4^, which fits our perception of nodal involvement as a marker of tumor burden or disease aggressivity^5,6^. We were intrigued on how nodal classifications would be modified after neoadjuvant chemotherapy and started gathering individual patients' data from the Universitair Ziekenhuis Brussel (UZ Brussel) twelve years ago to study the lymph node quanta in that context. For various reasons, completion of the study was delayed.

On the second facet, our concern has been the survival of patients. The past years has seen a growing literature recommending the restricted mean survival time (RMST) as a preferred measure to report survival results^7–10^. Global metrics like the hazard ratio or a logrank value are not readily obvious for patient's communication^11^. RMST, aka the expectation of life up to a given limit, is expressed as a time duration, e.g. a number of years out of a time horizon of, say 25 years. RMST is a clear intuitive outcome measure, understandable to patients^10^.

On the third facet, how to compare the outcomes, the RMST literature and available software have been limited to the comparison of two groups^10^. We used the lymph node ratio with RMST in a recent study^12^; comparisons had to reduce to a single 0.20 cutoff. Reducing to two groups did the job in the context of that study, but would not be satisfying in a formal comparison of classifications of four or more pre-established groups. Searching along Royston and Sauerbrei's concept of prognostic separation^13^, Gini's mean difference (GMD), the mean of absolute difference between all distinct pairs of quantities^14,15^, was found to express well the concept of separation. Application of the GMD to RMST has been presented for the first time in a study with disease-free survival endpoint from the Seoul National University Hospital (SNUH)^16^.

Meanwhile, a dozen years elapsed. Follow-up data from the UZ Brussel has matured, providing the unique opportunity to investigate the impact of nodal quanta on truly long-term overall survival.

## Results

### Patients

Out of 309 records of women who received primary chemotherapy for breast cancer in 1980–2009 at the radiotherapy department of the Universitair Ziekenhuis Brussel, N = 233 representing the study population had been diagnosed with histologically confirmed non-metastatic primary breast carcinoma. They underwent surgery with axillary lymph node exploration and had complete counts of lymph nodes. The data retrieval was finalized in February 2021. The median follow-up of patients then alive (N = 96) was 14.2 years (inter-quartile range [IQR] 11.4–19.1, minimum–maximum 0.6–34.7 years). The median age at diagnosis was 52.3 years (IQR 46.2–62.9). The median number of lymph nodes examined (*ntot*) were 14 (IQR 10–19), median number of positive nodes (*npos*) 2 (IQR 0–7), median lymph node ratio (*npos/ntot*) 0.18 (IQR 0.00–0.58). Other patients’ characteristics are summarized in Table 1. A high proportion of the patients were estrogen receptor (ER) and progesterone receptor (PR) negative, representing 46.2% of the 197 non-missing receptor status. Most patients had advanced initial T-stage tumor, 84.4% T3–T4, and had clinically involved lymph nodes prior to surgery, 58.8% N1-3. Most received mastectomy, 87.6%. Adjuvant radiation therapy (RT) was standard, 97.4% received RT after surgery. RT fields included the axillary-supraclavicular region in 78.2% and the internal mammary (IM, parasternal) in 33.3% of the patients. Post-operative chemotherapy was given to 27.5% of the patients and hormone therapy to 64.7%.

**Table 1:** Patient characteristics; N = 233.

Characteristic	Node-negative	Node-positive	*P*	Characteristic	Node-negative	Node-positive	*P*
	(N = 75)	(N = 158)					
Year of diagnosis			0.128	N nodes examined			0.574
1980–89	12 (16.0%)	34 (21.5%)		(0,9]	16 (21.3%)	39 (24.7%)	
1990–99	25 (33.3%)	66 (41.8%)		> 9	59 (78.7%)	119 (75.3%)	
2000–09	38 (50.7%)	58 (36.7%)		N positive nodes			< .001
Age at diagnosis			0.089	0	75 (100.0%)	0 (0.0%)	
≤ 45 years	21 (28.0%)	26 (16.5%)		(0,3]	0 (0.0%)	61 (38.6%)	
(45, 65 years]	42 (56.0%)	95 (60.1%)		(3,9]	0 (0.0%)	55 (34.8%)	
> 65 years	12 (16.0%)	37 (23.4%)		> 9	0 (0.0%)	42 (26.6%)	
Laterality			0.408	Lymph node ratio			< .001
Right (1 bilateral)	39 (52.0%)	73 (46.2%)		0	75 (100.0%)	0 (0.0%)	
Left (2 bilateral)	36 (48.0%)	85 (53.8%)		(0,0.2]	0 (0.0%)	48 (30.4%)	
Quadrant			0.010	(0.2,0.65]	0 (0.0%)	56 (35.4%)	
Outer	39 (57.4%)	69 (46.3%)		(0.65,1]	0 (0.0%)	54 (34.2%)	
Central	11 (16.2%)	54 (36.2%)		Surgery			< .001
Inner	18 (26.5%)	26 (17.4%)		Lumpectomy	24 (32.0%)	5 (3.2%)	
Histological grade			0.174	Mastectomy	51 (68.0%)	153 (96.8%)	
G1-2	22 (38.6%)	57 (49.6%)		Radiotherapy (RT)			0.450
G3-4	35 (61.4%)	58 (50.4%)		No	1 (1.4%)	5 (3.2%)	
Ductal component			0.930	Yes	69 (98.6%)	153 (96.8%)	
No	12 (16.0%)	26 (16.5%)		RT axillary-supraclav			< .001
Yes	63 (84.0%)	132 (83.5%)		No	47 (66.2%)	3 (1.9%)	
In situ component			0.783	Yes	24 (33.8%)	155 (98.1%)	
No	66 (88.0%)	137 (86.7%)		RT parasternal			< .001
Yes	9 (12.0%)	21 (13.3%)		No	56 (83.6%)	80 (58.4%)	
Neu status			0.367	Yes	11 (16.4%)	57 (41.6%)	
Negative	29 (67.4%)	54 (59.3%)		RT boost			< .001
Positive	14 (32.6%)	37 (40.7%)		No	48 (65.8%)	140 (89.2%)	
ER PR status			0.117	Yes	25 (34.2%)	17 (10.8%)	
ER– PR–	37 (57.8%)	54 (40.6%)		Chemotherapy			0.038
ER + PR–	6 (9.4%)	21 (15.8%)		Preop	61 (81.3%)	108 (68.4%)	
ER– PR +	3 (4.7%)	13 (9.8%)		Preop and postop	14 (18.7%)	50 (31.6%)	
ER + PR +	18 (28.1%)	45 (33.8%)		Preop chemo cycles			0.671
Preop T-stage			< .001	≤ 6	63 (91.3%)	127 (89.4%)	
T0-2	21 (28.4%)	15 (9.6%)		> 6	6 (8.7%)	15 (10.6%)	
T3	29 (39.2%)	59 (37.8%)		Anthracycline chemo			0.133
T4	24 (32.4%)	82 (52.6%)		No	9 (12.2%)	31 (20.3%)	
Preop N-stage			< .001	Yes	65 (87.8%)	122 (79.7%)	
N0	44 (59.5%)	50 (32.5%)		Taxane chemo			0.119
N1-3	30 (40.5%)	104 (67.5%)		No	51 (68.9%)	120 (78.4%)	
Postop T-stage			< .001	Yes	23 (31.1%)	33 (21.6%)	
ypT0-is	18 (24.7%)	3 (1.9%)		Hormone therapy			< .001
ypT1	23 (31.5%)	18 (11.5%)		No	40 (57.1%)	39 (25.3%)	
ypT2	18 (24.7%)	53 (34.0%)		Tamoxifen	23 (32.9%)	91 (59.1%)	
ypT3	7 (9.6%)	31 (19.9%)		Aromatase inhibitor	6 (8.6%)	23 (14.9%)	
ypT4	7 (9.6%)	51 (32.7%)		Else	1 (1.4%)	1 (0.6%)	

### Univariate analysis

**Figure 1:**
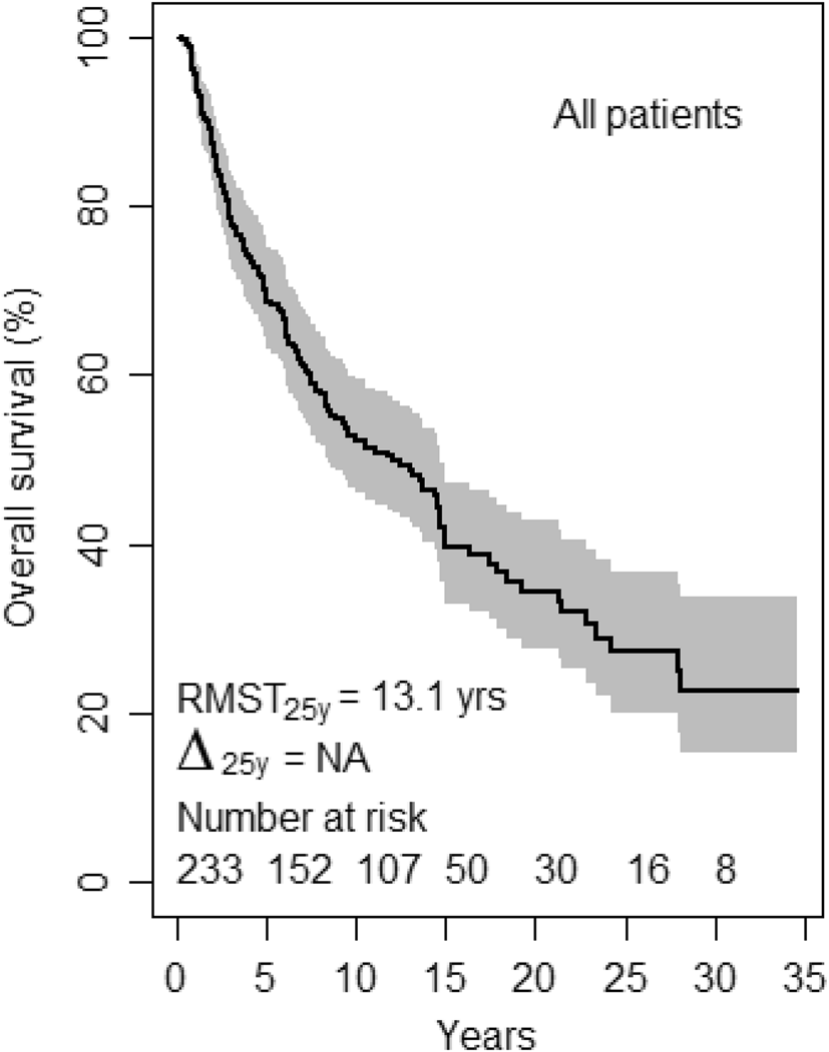
Overall survival, all patients. RMST_25y_, restricted mean survival time at 25 years time horizon. Δ_25y_, Gini's mean difference of the RMST. NA, not applicable.

The survival of the whole patients' population irrespective of characteristics was 13.1 years (standard error: ± 0.7) (Fig. 1). Note, here and throughout this report, "survival" not otherwise specified refers to the restricted mean survival time (RMST) and its standard error, computed with regard to a unique time horizon of 25 years, on a follow-up counted from the date of diagnosis to the date of death from any cause (event), or the date last known alive if no event occurred (censored).

**Figure 2:**
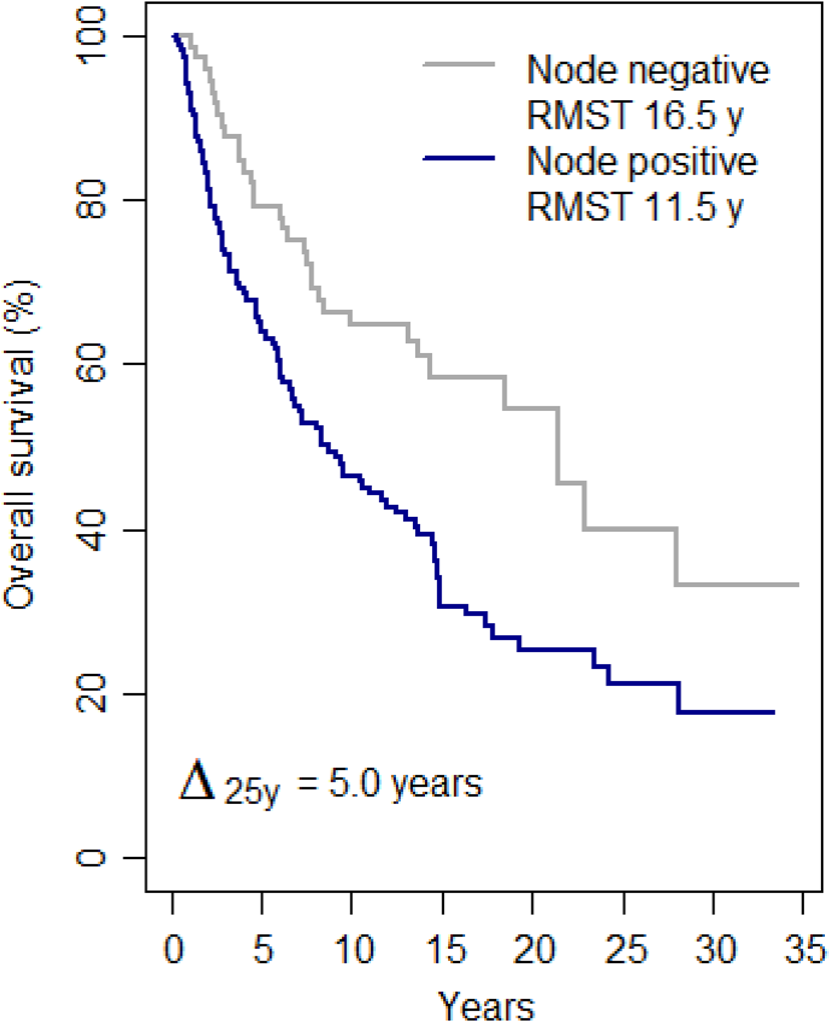
Survival according to post-chemotherapy pathological nodal status. RMST, restricted mean survival time at 25 years time horizon. Δ_25y_, Gini's mean difference of the RMSTs.

The survival according to the surgical pathology nodal status (Fig. 2) was 16.5 (± 1.1) years among node-negative patients (of whom 33 died) and 11.5 (± 0.8) years among node-positive patients (of whom 104 died). The Gini's mean difference Δ_25y_ (average of the absolute differences between all pairs of RMSTs, which in the case of two groups is the simple absolute difference) between the node-negative and node-positive groups was 5.0 (± 1.4) years.

**Figure 3:**
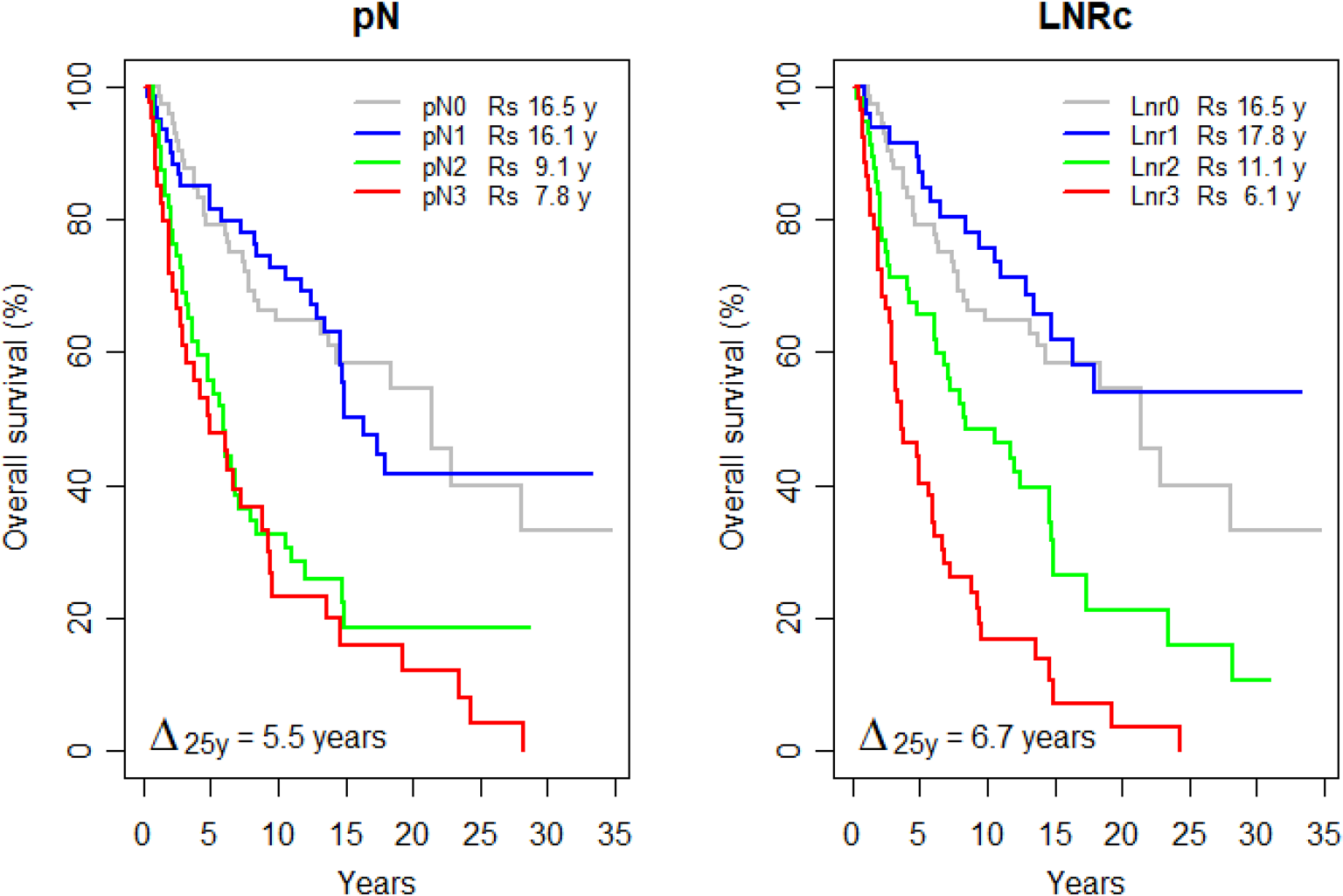
Survival according to nodal quanta classes. Rs, restricted mean survival time (RMST) at 25 years time horizon. Δ_25y_, Gini's mean difference of the RMSTs.

Figure 3 summarizes the survival according to the nodal quanta classes. The pN classification (Fig. 3 left graph; Table 2) appeared to identify only two patterns of survivors, one pattern with pN0 and pN1 showing similar survivals of 16.5 (± 1.1) and 16.1 (± 1.2) years, the other pattern with pN2 and pN3 showing similar (non-significantly distinct) survivals of 9.1 (± 1.2) and 7.8 (± 1.3) years, respectively. The Gini's mean difference Δ_25y_ for pN was 5.5 (± 0.9) years, not significantly different from the previous difference Δ_25y_ of 5.0 (± 1.4) years between node-negative and node-positive, suggesting that 4-groups pN did not separate much better than 2-groups.

**Table 2:** Univariate metrics of the nodal quanta classes.

Metric	Global pN	pN0	pN1	pN2	pN3	Global LNRc	Lnr0	Lnr1	Lnr2	Lnr3
Crude restricted mean survival time (RMST) at 25 years horizon (years)		16.5	16.1	9.1	7.8		16.5	17.8	11.1	6.1
Standard error of the RMST (years)		1.1	1.2	1.2	1.3		1.1	1.3	1.2	0.9
Gini's Δ25y of the crude RMSTs (years)	5.5					6.7				
Bootstrap standard error of the Gini's Δ25y (years)	0.9					0.8				
Crude log hazard ratios (HR) of the nodal classes		0 (Ref)	0.01	1.02	1.21		0 (Ref)	–0.26	0.73	1.45
Standard error of the nodal log HR		NA	0.255	0.237	0.246		NA	0.293	0.236	0.233
Akaike information criteria (AIC)	1313.4					1299.0				
Nagelkerke index of explained variation (R2N)	0.156					0.206				
Royston-Sauerbrei's measure of separation (D)	0.861					1.145				
Royston-Sauerbrei's index of separation (R2D)	0.150					0.238				
Concordance index (C)	0.641					0.668				
Harrell's *g*-index (*g*)	0.552					0.709				
Net reclassification improvement at 25 years (NRI)	0.319					0.331				

Regarding the LNRc classification, Fig. 3's right graph and Table 2 identified 3 distinct patterns of survivors: one pattern Lnr0 and Lnr1 showing similar survivals of 16.5 (± 1.1) and 17.8 (± 1.3) years, respectively, a second pattern with Lnr2 survival of 11.1 (± 1.2) years, and a third pattern with Lnr3 survival of 6.1 (± 0.9). The Gini's mean difference Δ_25y_ for LNRc was 6.7 (± 0.8) years, suggesting that LNRc might separate better.

Table 2 summarizes the univariate metrics to evaluate the nodal classifications. The top rows about the survivals (RMSTs) have been detailed above. The other measures—hazard ratios of the nodal classes, Akaike information criteria (AIC), Nagelkerke index of explained variation (R2N), Royston-Sauerbrei's measure of separation (D), Royston-Sauerbrei's index of separation (R2D), Concordance index (c), and Harrell's *g*-index—were computed from a Cox regression model with a single covariate, either the pN or the LNRc class, respectively. The net reclassification improvement at 25 years (NRI) was computed by comparing either pN or LNRc, respectively, with a dummy random variable unrelated to the present data. The metrics shown in the columns "Global pN" and "Global LNRc" evaluate the overall value of the corresponding nodal classification. All metrics—smaller AIC and larger R2N, D, R2D, C, *g*, and NRI– concurred with a better LNRc Δ_25y_.

### Multivariate analysis

The patients' items from Table 1, complemented with imputation of missing data, were used to search for a prognostic index model that did not include the nodal quanta classes. The variables as selected from a Cox stepwise regression by Akaike information criteria are shown in Table 3. All variables are self-explaining, except age, categorized into three groups but coded as a binary covariate, middle age (45, 65], coded 1, vs. younger ≤ 45 or older > 65, coded 0, based on the functional form of age^17^, detailed in the master thesis in Supplemental Material. The prognostic index was computed from the log of the hazard ratios (column P.I. HR imputed). The stability of the selection was evaluated through the percentage retained from AIC bootstrap resampling (column %bootstrap selected). The model was checked on the original non-imputed data to ascertain that there was no untoward discrepancy (column HR non-imputed). Lastly, the hazard ratios were compared with the confounder model published from the data set from the Seoul National University Hospital (column SNUH)^16^.

**Table 3:** Prognostic index (P.I.) survival model without nodal quanta.

Characteristic	Modeling	P.I. HR imputed	95% CI	% bootstrap selected	HR non-imputed	HR SNUH
Aromatase inhibitor	Binary	0.27	(0.11, 0.66)	99	0.49	0.51
Postop T-stage (ypT)	Ordinal	1.37	(1.14, 1.64)	97	1.46	1.94
Progesterone receptor	Binary	0.62	(0.42, 0.91)	93	0.53	0.66
Preoperative N-stage	Ordinal	1.51	(1.16, 1.98)	91	1.70	1.44
Parasternal radiation	Binary	0.65	(0.43, 1.00)	79	0.59	NA
Postoperative chemotherapy	Binary	0.65	(0.43, 1.00)	70	0.47	NA
Radiation therapy	Binary	0.43	(0.18, 1.03)	65	0.42	0.43
Age (45, 65 years]	Binary	0.72	(0.51, 1.02)	60	0.69	NA
Pathological Tumor size (cm)	Continuous	1.06	(0.99, 1.14)	54	1.06	NA

*HR* hazard ratio, *SNUH* Seoul National University Hospital, *NA* not applicable.

The check of the proportional hazards assumption using Schoenfeld residuals of the Table 3's prognostic index model showed significant departure for the Progesterone receptor and the Age (45, 65] covariates, *P* < 0.001 and *P* = 0.009, respectively, and a trend to departure for the Aromatase inhibitor covariate, *P* = 0.089. However, the prognostic index derived from Table 3 showed acceptable proportionality, *P* = 0.660.

The prognostic values of the nodal pN and LNRc classifications were evaluated in Cox proportional hazard models stratified on quartiles of the prognostic index. The subsequent metrics are shown in Table 4. Qualitatively, the multivariate metrics mirror the univariate metrics. Even though not as markedly as shown by the univariate metrics, overall the LNRc classification consistently improved on the pN classification.

**Table 4:** Multivariate metrics of the nodal quanta classes.

Metric	P.I. with pN					P.I. with LNRc				
	Global pN	pN0	pN1	pN2	pN3	Global LNRc	Lnr0	Lnr1	Lnr2	Lnr3
Adjusted RMST at 25 years horizon (years)		16.0	16.9	9.4	9.6		16.0	18.1	11.3	9.1
Standard error of the RMST (years)		1.4	1.5	0.6	0.7		1.4	1.7	0.9	0.6
Gini's Δ25y of the adjusted RMSTs (years)	4.8					5.3				
Bootstrap standard error of the Δ25y (years)	0.8					0.9				
Log Hazard ratio (HR) of the nodal classes		0 (Ref)	−0.15	0.90	0.87		0 (Ref)	−0.34	0.66	0.95
Standard error of the nodal log HR		NA	0.257	0.246	0.256		NA	0.294	0.238	0.248
Akaike information criteria (AIC)	924.1					923.2				
Nagelkerke index of explained variation (R2N)	0.112					0.115				
Royston-Sauerbrei's measure of separation (D)	0.377					0.618				
Royston-Sauerbrei's index of separation (R2D)	0.033					0.084				
Concordance index (C)	0.619					0.631				
Harrell's *g*-index (*g*)	0.496					0.547				
Net reclassification improvement at 25 years (NRI)	0.338					0.364				

Larger metric value indicates better prognostication, except the Akaike Information Criteria (AIC) for which smaller is better. P.I., prognostic index model as detailed in Table 3.

*NA* not applicable.

### Node-negative

**Figure 4:**
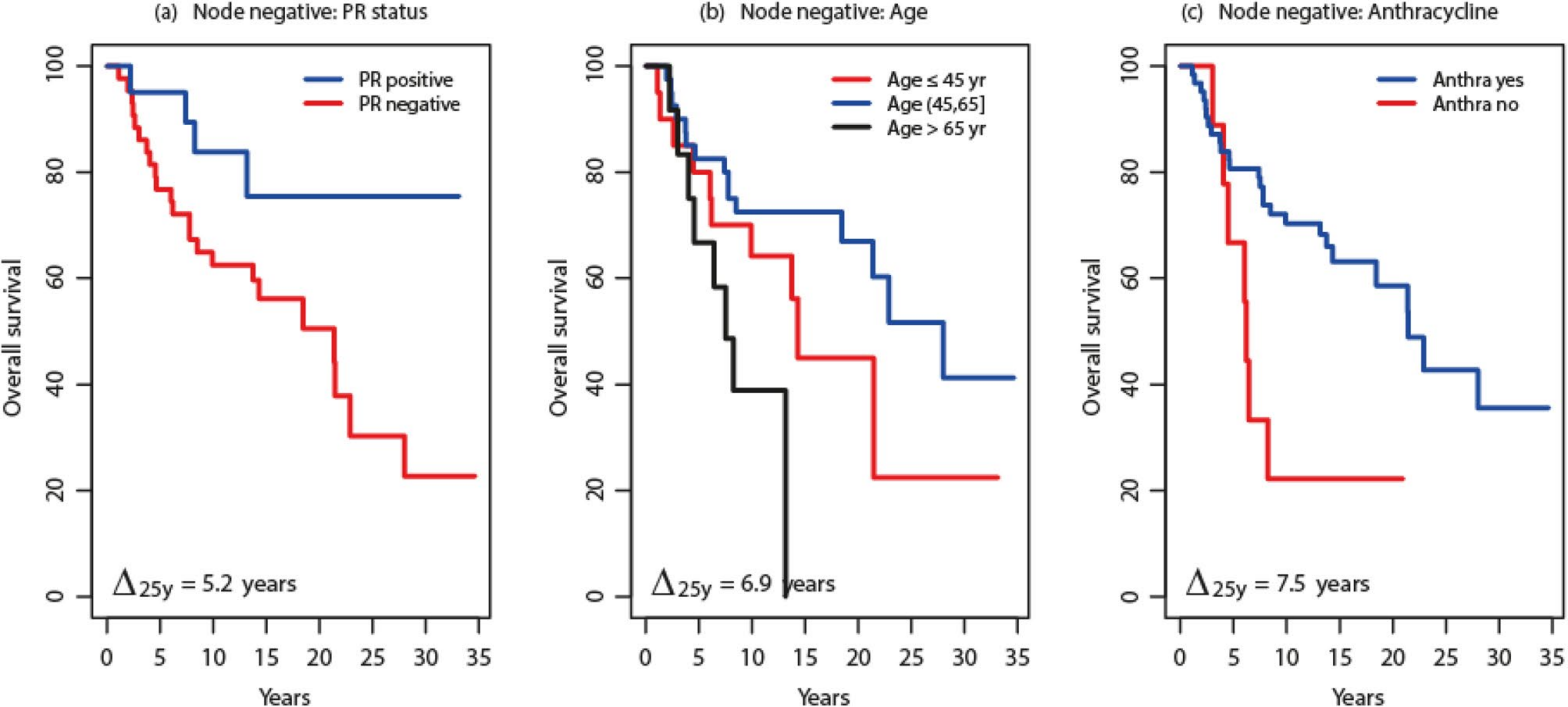
Survival among post-chemotherapy node-negative patients according to (**a**) progesterone receptor (PR), (**b**) age 45–65 years, and (**c**) receipt of anthracycline chemotherapy. Δ_25y_, Gini's mean difference of the restricted mean survival times at 25 years time horizon.

The similarity between the survival of node-negative patients and pN1 or Lnr1 was intriguing. Browsing the data on multiple factors identified several potential indicators of prognostic heterogeneity among the node-negative patients. These are summarized in Fig. 4.

According to progesterone receptor (PR) status, PR negativity among the N = 75 node-negative patients was associated with a survival of 15.8 (± 1.4) years, whereas PR positivity was associated with a distinctly better survival of 20.9 (± 1.8) years, representing a Gini's difference Δ_25y_ of 5.2 (± 2.2) years. It is worth reminding that the time horizon to compute survival was defined at 25 years. This puts the 20.9 years survival of the node-negative PR-positive group into a near-cure perspective.

According to age at diagnosis, younger age ≤ 45 years old was associated with a survival of 14.8 (± 2.1) years, middle age > 45 to 65 years old was associated with a survival of 18.7 (± 1.4) years (best of the three groups), and older age > 65 years old was associated with a survival of 8.3 (± 1.2) years (poorest of the three groups). The survivals were significantly distinct, with a Gini's mean difference Δ_25y_ of 6.9 (± 1.2) years.

According to chemotherapy with or without anthracycline, receipt of anthracycline was associated with a survival of 17.4 (± 2.7) years. Non-anthracycline therapy was associated with a significantly poorer survival of 9.8 (± 1.2) years, the Gini's mean difference Δ_25y_ was 7.5 (± 3.0) years.

## Discussion

1. There is nothing new. Much of the hard work that led to the discovery of the importance of the lymph node ratio in breast and other cancers was accomplished twenty years ago. In-depth analyses of nodal quanta were done on 37′519 node-negative and 16′978 node-positive breast cancer cases, covering the number of uninvolved nodes, the number of negative nodes, the number of positive nodes^1^. The functional form of these quanta showed their non-linearity and considerable overdispersion^2^. An analysis of 83,686 cases of T1–T2 breast cancer demonstrated the fundamental property of the lymph node ratio as a better behaved truly linear variable and able to obviate the overdispersion of the other quanta^3^. The first systematic review of the literature conducted by the non-profit International Nodal Ratio Working Group (INRWG) in 2006 pooled 31′879 patients from 23 independent clinical studies, establishing the prognostic value of nodal ratios in breast cancer^18^. It also acknowledged the precedence of Fletcher and Montague in 1980^19^. A compiled update pooling 111′829 patients further confirmed the value of the nodal ratios in practically all related areas^20^: (1) identifying high-risk subgroups of patients for adjuvant locoregional therapy; (2) impact on radiation treatment volume decisions; (3) role in neoadjuvant therapy; (4) the maintained value in micrometastatic nodal involvement; (5) value in locally advanced disease; (6) value in distant metastatic stage; (7) interaction with age. A bootstrap regression study from Geneva, Switzerland, introduced the 0.20, 0.65 cutoffs^21^. Numerous other studies have confirmed the superiority of the lymph node ratio and the cutoffs; we cite non exhaustively from the US, Korea, Netherlands, Italy, China, Turkey, in diverse conditions such as triple negative breast cancer^22–28^.
2. There is neither anything new in the RMST. The term "restricted mean survival time" was introduced by Andersen in 2004^29^. The term was new, but not what it represented. The author credited the functional to Irwin who used in 1949 the term "expectation of life limited to *n* years"^30^. Even then, it was not new, the method had been implemented in 1946 by the same author in collaboration with Goodman^31^. Twenty years before that, but not cited by Irwin, Greenwood in an experiment on a herd of mice computed the "expectation of life limited to 60 days"^32^. Back forward, in one of the topmost cited papers^33^, Kaplan and Meier in 1958 devoted a section of the Product-Limit estimate to the Mean lifetime^34^. They noted *if the probability of an indeterminate result is high, there is no satisfactory way to estimate µ* (the mean), upon which Irwin's approach was acknowledged as a solution, *that in place of estimating the mean itself, one should estimate the "mean life limited to a time L," say µ(L)*, with *L chosen at the investigator's convenience*, and advised *one would choose L to make the probability of an indeterminate result quite small*^34^*,* an issue still discussed nowadays. The importance did not escape notice as reported by Armitage in 1959, Meier pointed out that the *mean life limited to a time T* was an alternative actuarial method to compare survival curves^35^. Thus, the paper trail shows RMST fundamentals were well established more than 60 years ago.
3. Gini's mean difference is even older: in 1912, Corrado Gini presented his monography on the variability and mutability contributing to distributions and statistical relations, within which the GMD (Δ) was formally established as *la differenza media tra più quantità*, i.e. the average difference between multiple quantities ^14^. Gini derived in 1914 an index that scaled Δ with twice the mean value of the quantities from which Δ was computed, *G* = Δ/(2μ)^36^. The unitless *G* index, also called Gini coefficient or Gini index, is widely recognized in numerous domains, ranging from social sciences to mathematical physics^37–44^, whereas Δ, expressed in the same units as its computing quantities, has been rediscovered^45,46^, and, since 2015 has been integrated in a major statistical modeling package^15^. Thus, as with RMST, GMD is neither new nor forgotten.
4. The novelty is in combining the components, in applying the GMD to the RMST to create a new measure. At the time of this writing, a search of Pubmed finds out of 33 million articles only one paper that applied GMD as a survival metric^16^. The GMD of the RMST provides a fresh perspective in survival analysis. The present study of survival after neo-adjuvant chemotherapy shows, very simply, what are the information gained or lost with long-term follow-up when using either the pN or the LNRc classification. Overall LNRc outperformed pN, but without implying that pN should be dismissed (Fig. 3, Table 4).
5. The relatively good prognosis of Lnr1, and also pN1, is in line with the bulk of earlier studies (already cited at the start of the Discussion) that identified a lymph node ratio ≤ 0.20 as low risk. We remind that patients were treated in 1980–2009, era of full axillary lymph node dissection. Most patients had > 9 lymph nodes examined; the median was 14 nodes. This implies that the 1 to 3 positive nodes pN1 almost matches Lnr1, with very few pN1 patients with a ratio > 0.20. Consequently, the pN1 survival of 16.1 (± 1.2) years is quite well in keeping with the present Lnr1 survival of 17.8 (± 1.3) years (it is easily seen that any value fully falls within 2 standard errors of the other value).
6. The relatively poor survival of node-negative cases is an unexpected finding. In an earlier version of the study, we attributed this to differences in *ntot*. But node-negative patients had the same extent of lymph node examination as node-positive. We questioned whether the quite poor survival was instead due to heterogeneity among node-negative patients, due to differences in tumor biology, in patient's characteristics, or in therapy. With only 75 node-negative patients of whom 33 died, it is not realistic to expect that the present data can elucidate. Nevertheless, Fig. 4 suggests that any of the three shown factors, or a combination thereof, can be a potential cause. A study of 4′453 women with breast cancer from the Malmo University, Sweden, found that women < 40 years old had a poor prognosis, the association with age was strongest among node-negative patients^47^. A study from Stanford, California, of 220 women aged ≤ 40 years old who underwent neoadjuvant chemotherapy observed that those who achieved a pathologic response in the lymph nodes but had residual disease in the breast continued to have outcomes similar to those who remained node-positive^48^. These studies and Fig. 4 hint at a possible interaction with age, further investigation with more node-negative patients is needed.
7. We have reflected that axillary lymph node involvement at the moment of surgery did not occur overnight but is a snapshot of a disease that evolved over time^6^. Over a decade ago we considered nodal ratios as a bridge to biomarker staging, notably with circulating tumor cells^20^, e.g. dynamic instead of static assessment. An alternative approach no less dynamic than circulating tumor cells or liquid biopsies based on PET scan might be considered. Recently finalizing a long-term follow-up re-analysis study of preoperative positron emission tomography (PET), we observed that like other biomarkers, positive axillary PET was predictive of early^49^, not for late disease-free survival (non-proportionality of the hazard)^50^, but was a predictor of overall survival at 15 years^50^. The study identified the ratio of *ipsilateral* axillary maximum standard uptake value (SUVmax) over the *contralateral* axillary SUVmax as the strongest predictor of 15 years survival. The PET study is scheduled to be expanded with an updated cohort of patients identified in 2009–2015^51^. The relevance to the present report is on the following points: (1) the prognostic value of ipsilateral/contralateral axillary SUVmax if confirmed will pave the way to a new type of nodal quanta, noninvasive and repeatable; (2) axillary surgery changed from dissection to sentinel biopsy, the updated study will inform on the prognostic stability or not of the surgical nodal quanta; (3) more patients with less advanced disease underwent neoadjuvant chemotherapy, which will allow to evaluate how outcomes are affected as compared to the present report. Although delayed for Covid and lack of funding, we have no doubt that the new planned study will someday come to completion.
8. Limitations of the present study includes its retrospective nature. Despite the utmost care given to abstraction and data analysis (master thesis in Supplemental Material), source errors and miscoding transcriptions are inherent. Small study size restricts the possibility of more advanced modeling. Treatments occurred over a long period, staging and management changed over time. Other weaknesses are highlighted in comparison with the published SNUH study, which had systematic advanced preoperative imaging and biological markers, much of which were missing in our dataset. Quality of life of patients were not assessed. There are some weaknesses in the GMD. It does not indicate the direction of the differences, but that is quite minor, a look at RMST would immediately show which values are larger. With only 3 groups, GMD depends only on the two extremes. GMD is not affected by ordering of the groups, but whether that is a weakness or not will have to be investigated.
9. Strengths include the long follow-up with a large number of events that ensured maturity of the survival data. More info on radiation therapy were available. Learning from the earlier collaboration with SNUH, data analyses were enhanced, allowing a streamlined approach.
10. In summary, Gini's mean difference of restricted mean survivals represents a new tool that streamlines survival analyses. In a comparison of nodal quanta, number versus ratio of positive nodes over the number of examined nodes, at a time horizon of 25 years, a ratio-based classification displayed a better prognostic separation than numbers. An unexpected finding was the relatively poor outcome of node-negative patients after neoadjuvant chemotherapy that will require further investigation. Future perspectives will be the study of alternative noninvasive nodal quanta, such as could be provided by circulating biomarkers or by metabolic imaging.

## Materials and methods

We retrieved the records of women who had been referred for treatment between 1980 and 2009 to the radiotherapy department of the Universitair Ziekenhuis Brussel (UZ Brussel). Patients were selected according to the following criteria: women diagnosed with a histologically confirmed non-metastatic primary breast carcinoma who underwent surgery of the breast with axillary lymph node exploration, in whom chemotherapy was given prior to surgery. Records of patients without information on number of positive nodes and number excised were excluded. Age was not used for selection.

Randomization was not performed. The study retrospectively collected non-experimental data already recorded in charts. Informed consent to participate was waived and approved by the Universitair Ziekenhuis Brussel (UZ Brussel). All methods were carried out in accordance with relevant guidelines and regulations. All experimental protocols were approved by the Institution Review Board of the Universitair Ziekenhuis Brussel.

### Data

The data abstracted were: patient’s age, menopausal status, dates of diagnosis, surgery, follow-up and occurrence of events, tumor laterality, tumor location, histopathology, histological grade, neu status, estrogen receptor (ER) and progesterone receptor (PR) status, preoperative T, N, and M stage; type of preoperative chemotherapy, number of chemotherapy courses; type of surgery, tumor size assessed by surgical pathology, number of lymph nodes examined (*ntot*), number of positive nodes (*npos*); postoperative radiotherapy given or not, type of radiotherapy equipment, doses delivered, whether treatment fields included the internal mammary and/or axillary-supraclavicular regions; adjuvant chemotherapy, and adjuvant hormone therapy. For patients with bilateral tumors, only the first chronological entries were abstracted.

The specific organization of the patient's database, the coding, the extraction procedure, and the related computational details are provided in the master thesis in Supplemental Material.

The number-based classification pN assigned *npos* of 0, 1–3, 4–9, and 10 + , to pN0, pN1, pN2, and pN3, respectively. The ratio-based classification LNRc assigned ratios *npos/ntot* of 0, (0, 0.20], (0.20, 0.65], and (0.65, 1.00], to Lnr0, Lnr1, Lnr2, and Lnr3, respectively^21^. We used the term "nodal quanta classes", *quanta* short for quantified, to bring to the fore that in this study "pN" and "Lnr" are labels for numerical quantities. Clinical-pathological classifications such as N-stage mix numerical classes with several different qualitative classifiers such as fixed, matted, internal mammary; the study used the mixed clinical ordinal N-stage as a covariate.

### Survival analysis

The endpoint was overall survival, from time of diagnosis to event defined as death from any cause, or last known follow-up if no event occurred. Survival curves were established using the Kaplan–Meier method^34^, and modeling used the Cox proportional hazard^52^. The restricted mean survival time (RMST), computed for a time horizon of 25 years, was used as the study main measure of survival, in accordance with actual recommendations^7,8^. As already mentioned, RMST is the expected remaining life from a time origin to a specified time horizon, discounting future years beyond the horizon^9,53^.

### Gini's mean difference, a new metric of prognostic value

Until now, usage of RMST in the literature has been limited to the comparison of two groups, either by the difference of RMST between the groups^9^, or by the ratio between the two RMST^10,53^. Regardless of any advantage, limitation to two groups would curb the role of RMST, as clinical studies may require to investigate considerably more than only two groups^54^. Fortunately, the limitation has just been lifted. Authors from Korea and Martinique demonstrated that Gini's mean difference (GMD) was applicable to analyze the RMSTs of any number of groups^16^. They showed that the GMD applied to RMST generalized the 2-groups comparison.

For a set of *n* "observations" *X*_*1*_*, …, X*_*n*_, the Gini's mean difference Δ is the mean absolute difference between any two distinct "observations"^15,16^:

$$\Delta = \frac{1}{{n\left( {n - 1} \right)}}\mathop \sum \limits_{{\begin{array}{*{20}c} {i,j = 1} \\ {i \ne j} \\ \end{array} }}^{n} \left| {X_{i} - X_{j} } \right| .$$

It is a measure of dispersion that reflects the *disparity* of the "observations". The more separation there is between the outcomes of groups defined by a prognostic marker, the more disparity there is, the better it is for the prognostic marker, the more value it has. The GMD was used in the present report as the main metric to compare the nodal quanta classifications.

GMD applied to RMST is new, there is only one precedent study^16^. The other better-known metrics have been presented to facilitate balancing this study with the literature: the Akaike information criterion (AIC) indicator of model quality^55^, the Nagelkerke's R2N measure of explained randomness in a model^56^, the Royston and Sauerbrei's D measure, where D is a log hazard ratio that quantifies the prognostic separation between subjects with low and high predicted risk^13^, the R2D derived from D as an index of separation^13^, the C index of the probability of concordance based on rankings^15^, the Harrell's *g*-index, a new measure of a model’s predictive discrimination based on the GMD of the model's linear predictors^15^, and the net reclassification improvement (NRI)^57^.

All statistical analyses used version 4.1.2 of the R project^58^. The AIC, Gini’s mean difference and its bootstrap standard error were computed using in-house scripts. The net reclassification improvement used the package *survIDINRI*^57^. Harrell's *g*-index was computed with the function *cph* of the package *rms*^15^. Computation of RMST and other metrics used the 2021's version of the package *survival*^52^.

### Consent for publication

All authors consented.

## Supplementary Information

Supplementary Information.

## Data Availability

Data and software will be made available through https://doi.org/10.17632/7vpg85kxsm.1 .
