# Supplementary material for: Gini's mean difference and the long-term prognostic value of nodal quanta classes after pre-operative chemotherapy in advanced breast cancer
Source: Sci Rep. 2022 Feb 22;12:2983. doi: 10.1038/s41598-022-07078-7 (PMC8863879; doi:10.1038/s41598-022-07078-7)
Supplement: Supplementary file 1 — Supplementary Information. [file 41598_2022_7078_MOESM1_ESM.pdf]

Post-mastectomy radiation treatment in T1-T2 breast cancer

Vincent Vinh-Hung

Prof. Marc Nyssen

VUB

Eindwerk
Master na master in het beheer van gezondheidsgegevens
2004-2005

With grateful acknowledgment to Prof. Guy Storme for the original concept of the study, Mia Voordeckers for the first draft and the maintenance of the database, Cornelia Claassens, Marc Robberechts, Myriam Spinnoy, Prof. Jan Lamote, and many others who helped with the gathering of data.

Contents

Abstract Page 4
Section1. Introduction 5
Section 2. The SEER data 7
Section 3. AZ-VUB Breast Cancer data and selection 13
- 1. Description 13
- 2. Selection of patients 14
- 3. Variables and endpoints of interest 15
- 4. Imputation of partly missing tumour size 20
- 5. Imputation of partly missing date of diagnosis 23
- 6. Database difficulties 23
- 7. Final selection 24

Section 4. Methods 25
Section 5. Concordance SEER and AZ-VUB 27
Section 6. Comparison of SEER and AZ-VUB treatment outcome 31
Section 7. Discussion 38
Appendix 40
- A1. Structure of "Mamma juni 2005" database 41
- A2. Radiation treatment folder at the AZ-VUB 44
- A3. WHO histological classification 48
- A4. Histopathologic grade classification. 49
- A5. TNM classification 50

References 52

Abbreviations

AZ-VUB: Academisch Ziekehuis, Vrije Universiteit Brussel
BCS: Breast conserving surgery
npos: number of positive (involved) axillary lymph nodes
ntot: number of examined (excised, removed) axillary lymph nodes
PMRT: Post-mastectomy radiation treatment (radiotherapy)
RT: Radiation treatment (radiotherapy)
SEER: Surveillance, Epidemiology, and End Results

Abstract

The role of post-mastectomy radiation treatment (PMRT) in low or intermediate risk breast cancer, defined as small tumours with no or few involved axillary lymph nodes, is one of the most controversial issues in the local-regional management of breast cancer. Subgroup analyses of randomized clinical trials have been unable to clarify whether or not PMRT can improve survival. The present paper examines the potential contribution of the AZ-VUB, in whom most often mastectomy patients received radiation, as compared with the US SEER, in whom mastectomy patients infrequently received radiation. The respective databases are presented in detail to evaluate the adjustments required for merging the databases to allow a common modeling. The similarities of the patient populations are ascertained by verifying the concordance of prognostic factors. Thereafter, a formal comparison of survival outcomes is performed with regard to receipt or not of post-surgery radiation. By examining different contrasting combinations of the AZ-VUB and the SEER patients, the paper concludes that PMRT is associated with a substantial survival advantage in node-positive patients, regardless of the extent of nodal involvement.

Section 1. Introduction

In western countries, breast cancer is the most common cancer type in women and the incidence has been rising continuously (*1*). In Belgium 6628 new cases were reported in 1998, which corresponds with 35.5 % of all cancers in women. In 1997, 2416 deaths from breast cancer were reported (*2*). The primary treatment for small tumours is breast conserving surgery (BCS) or mastectomy (*3*). There is a general consensus about the survival benefit of adjuvant radiotherapy after BCS (*4*,*5*). The role of adjuvant radiotherapy after mastectomy is more controversial. It is generally admitted that post-mastectomy radiotherapy (PMRT) is beneficial in high risk patients (patients with large tumour (T3-stage, >5 cm) and/or with 4 or more than 4 positive axillary lymph nodes) (*4*,*6*). But for lower risk patients (small tumour, and node-negative or node-positive with <4 positive nodes), it has been considered that there is insufficient evidence to support the use of PMRT in these patients (*4*). In their investigation of the US Surveillance, Epidemiology, and End Results (SEER) population data, other authors also failed to find a survival benefit for PMRT in low risk patients (*7*,*8*). The criticism was however the retrospective nature of these population studies. Adverse selection factors could have masked any beneficial role of PMRT in the low-risk patients (*8*).

Since extensive meta-analyses have failed to provide an answer to the issue of PMRT in low-risk patients, there is a need to consider alternative investigation strategies. In our radiotherapy department at the Academisch Ziekenhuis, Vrije Universiteit Brussel (AZ-VUB) since its creation in 1984, node-positive patients have received PMRT regardless of the size of the primary tumour and regardless of the number of positive axillary lymph nodes. Unlike the SEER, treatment outcome of our patients would be unbiased by adverse selection. This hints at the possibility of making a comparative study between the SEER and the AZ-VUB.

Therefore, the primary objective of the present study is to compare the survival outcome of patients from the SEER and from the AZ-VUB, in order to gain insight into the role or lack of role of PMRT in low-risk breast cancer women (primary tumour <= 5 cm, node-negative or node-positive with 1 to 3 positive axillary lymph nodes).

It might seem paradoxical that while our department have extensively investigated breast cancer data from randomized clinical trials and from the SEER (*5*,*8*-*22*), fewer investigations have been done using our own data (*23*,*24*). This hints at problems of data registration and availability. Therefore, the secondary objective of the present study is to identify difficulties encountered in the investigation of our own data.

The structure of the paper is as follows. Section 2 describes the SEER data. Section 3 describes the AZ-VUB data and summarizes the data selection. Section 4 lists the statistical methods used. Section 5 addresses the issue of fundamental comparability of the SEER and the AZ-VUB data, namely, are findings on the effects

of lymph node involvement, tumour size, and age based on the SEER supported by the AZ-VUB data or not. Section 6 makes the comparisons of post-mastectomy survival outcome between the SEER and the AZ-VUB. Section 7 is a discussion.

Section 2. The SEER data

As shown in Table 1, the SEER is one among three US National Cancer Data Programs established to monitor cancer care (*25*).

Table 1. Characteristics of the three US Cancer Data Programs –NPCR, SEER, NCDB (*25*), and the AZ-VUB Breast Registry

Characteristic	NPCR National Program of Cancer Registries	SEER Surveillance, Epidemiology, and End Results	NCDB National Cancer Data Base	AZ-VUB Academisch Ziekenhuis, Vrije Universiteit Brussel, Breast Registry
Purpose	Surveillance	Surveillance	Quality of care	Treatment evaluation
Sponsor	Centers for Disease Control and Prevention	National Cancer Institute	American College of Surgeons' Commission on Cancer; American Cancer Society	None
Financial support*	$32 million per year (75% CDC; 25% State)	$22 million per year (80% NCI; 20% State)	$1.2 million per year	None
Geographic coverage	National, except 5 SEER states	Limited (in 2001: 9 states§ and 6 areas¶)	National	Hospital network Brussels, Vilvoorde, Aalst, Mechelen, Oostende
Population-based	Yes	Yes	No	No
Source of cases	Hospitals, MD offices/clinics, Pathology labs, Out-of-state registries, Death certificates	Hospitals, MD offices/clinics, Pathology labs, Out-of-state registries, Death certificates	Hospitals	Patients referred to AZ-VUB Radiotherapy and Surgery departments
Cases/records added annually	Roughly 1 million	170,000	873,000	200
Treatment data	First course only	First course only	First course, surgical detail, reconstructive procedures, biological response modifier therapy	First course, surgical detail, reconstructive procedures, radiation and systemic treatment detail
Data availability	Through NAACCR (North American Association of Central Cancer Registries)	Public-use files	No	No

* Costs associated with data collection are borne by the reporting facilities.
§ Connecticut, Iowa, New Mexico, Utah, Hawaii, California, Kentucky, New Jersey, Louisiana.
¶ Detroit, Seattle-Puget Sound, Atlanta, rural Georgia, American Indians in Arizona, Native populations in Alaska.

The SEER is a system of population-based tumour registries administered since 1973 by the National Cancer Institute (NCI). The NCI contracts with organizations in several states, metropolitan areas, and minorities registries, to collect information on all new cases of cancer diagnosed in their geographic areas (*25*). SEER began collecting data on cases on January 1, 1973, in the states of Connecticut, Iowa, New Mexico, Utah, and Hawaii and the metropolitan areas of Detroit and San Francisco-Oakland (*26*). In 1974-1975, the metropolitan area of Atlanta and the 13-county Seattle-Puget Sound area were added (*27*). These 5 states and 4 metropolitan areas are commonly referred to as the SEER "9-registries". The 9-registries represent approximately 10% of the US population. The SEER "11-registries" refers to the 9-registries, plus Los Angeles and San Jose-Monterey which joined the SEER program in 1992. The SEER "12-registries" refers to the 11-registries, plus the Alaska Native population, for whom data was registered from 1992. Rural counties in Georgia were added in 1978, and American Indians residing in Arizona in 1980. In 2001, the SEER program expanded coverage includes Kentucky, Louisiana, New Jersey, and the remainder of California, covering in total approximately 26 percent of the US population (*27*).

The SEER Registries routinely collect data on patient demographics, primary tumor site, morphology, stage at diagnosis, *first course* of treatment, and follow-up for vital status (Table 2) (*28*). The mortality data reported by SEER are provided from death certificates by the National Center for Health Statistics (*27*). Detailed data on tumour extension was recorded from 1988 (Table 3) (*29*).

Table 2. SEER public access record description (*28*).

Item	Item name	Item	Item name
01	SEER registry	40	Histology edit override
02	Case number	41	Age-site edit override
03	Record number	42	Sequence number - dx conf override
04	Type of reporting source	43	Site-type-lat-seq override
05	Place of birth	44	Surgery- diagnostic conf override
06	Year of birth	45	Report source sequence overrride
07	Age at diagnosis	46	Seq-ill defined site override
08	Race/ethnicity	47	Leuk-Lymph dx confirmation override
09	Spanish surname or origin	48	AJCC stage 3rd edition (1988+)
10	Sex	49	Tumor Marker 1
11	Marital status at diagnosis	50	Tumor Marker 2
12	Sequence number	51	ICD-O-2 Conversion flag
13	Year of diagnosis	52	Site-behavior override
14	Primary site	53	Site-EOD-diagnosis date override
15	Laterality	54	Site-laterality-EOD override
16	Histologic Type ICD-O-2	55	Site-laterality-morphology override
17	Behavior Code ICD-O-2	56	Recode ICD-O-2 to 10
18	Grade	57	ICCC site recode
19	Diagnostic confirmation	58	SEER modified ICCC site recode
20	Extent of disease (12 digit)	59	Site re with Kaposi and mesothelioma
21	Site specific surgery (1983-1997)	60	Race recode Y
22	Reason no surgery	61	Race recode Z
23	Radiation	62	Origin recode
24	Radiation to Brain and/or CNS	63	Cause of Death to SEER Site Recode
25	Radiation sequence with surgery	64	Tumor Marker 3
26	Vital status recode	65	Number of primaries
27	Histologic Type ICD-O-3	66	Surgery of Primary Site (1998+)
28	Behavior Code ICD-O-3	67	Scope of Regional Lymph Node Surgery (1998+)
29	Site recode	68	Number of Regional Lymph Nodes Examined
30	Race recode A	69	Surgery of Other Regional Site(s), Distant Site(s), or Distant Lymph Node(s)
31	Race recode B	70	Reconstruction–First Course
32	Age recode <1 year olds	71	First malignant primary indicator
33	SEER historic stage A	72	ICD-O Coding Scheme
34	SEER modified AJCC stage 3rd ed (1988+)	73	ICD-O-3 Conversion flag
35	SEER Summary Stage 1977 (1988+)	74	Behavior recode for Analysis
36	SEER Summary Stage 2000 (1998+)	75	COD to site rec KM
37	Survival time recode	76	State-county recode
38	Recode ICD-O-2 to 9	77	Type of follow-up expected
39	Site-type edit override		

Table 3. SEER Coding of extent of disease (*29*)

SIZE OF PRIMARY TUMOR (from pathology report; operative report; physical examination; mammography examination– in priority order; if multiple masses, code largest diameter)

Code	Description	
000	No mass; no tumor found; no Paget's disease	
001	Microscopic focus or loci only	
002	Mammography/xerography diagnosis only with no size given (tumor not clinically palpable)	
	mm	cm
003	<=3	<=0.3
...		
009	9	0.9
010	10	1.0
...		
099	99	9.9
100	l00	10.0
...		
990	990+	99.0+
997	Paget's Disease of nipple with no demonstrable tumor	
998	Diffuse; widespread: 3/4's or more of breast; inflammatory carcinoma	
999	Not stated	

EXTENSION

Code	Description
00	IN SITU: Noninfiltrating; intraductal WITHOUT infiltration; lobular neoplasia
05	Paget's disease (WITHOUT underlying tumor)
10	Confined to breast tissue and fat including nipple and/or areola
20	Invasion of subcutaneous tissue; Skin infiltration of primary breast including skin of nipple and/or areola; Local infiltration of dermal lymphatics adjacent to primary tumor involving skin by direct extension
30	Invasion of (or fixation to) pectoral fascia or muscle; deep fixation; attachment or fixation to pectoral muscle or underlying tissue
40	Invasion of (or fixation to) chest wall, ribs, intercostal or serratus anterior muscles
50	Extensive skin involvement: Skin edema, peau d'orange, "pigskin," en cuirasse, lenticular nodule(s), inflammation of skin, erythema, ulceration of skin of breast, satellite nodule(s) in skin of primary breast
60	(50) plus (40)
70	Inflammatory carcinoma, incl. diffuse (beyond that directly overlying the tumor) dermal lymphatic permeation or infiltration
80	FURTHER extension: Skin over sternum, upper abdomen, axilla or opposite breast
85	Metastasis: Bone, other than adjacent rib; Lung; Breast, contralateral–if metastatic; Adrenal gland; Ovary ; Satellite nodule(s) in skin other than primary breast
99	UNKNOWN if extension or metastasis

Note 1: Changes such as dimpling breast of the skin, tethering, and nipple retraction are caused by tension on Cooper's ligament(s), not by actual skin involvement. They do not alter the classification.

Note 2: Consider adherence, attachment, fixation, induration, and thickening as clinical evidence of extension to skin or subcutaneous tissue; code '20'.

Note 3: Consider "fixation, NOS" as involvement of pectoralis muscle; code '30'.

Note 4:	If extension code is:	Behavior code must be:
	00	2
	05	2 or 3
	10+	3

LYMPH NODES

0 No lymph node involvement

REGIONAL Lymph Nodes (ipsilateral). Axillary– Level I/low: Adjacent to tail of breast– Level II/mid: Central, interpectoral, (Rotter's node)– Level III/high: Subclavicular, apical– Infraclavicular– Intramammary– Nodule(s) in axillary fat.

Size of largest metastasis (Effective date January 1, 1992 diagnoses) in axillary node(s), ipsilateral (codes 1-4):

Code	Description
1	Micrometastasis (<=0.2 cm)
2	>0.2-<2.0 cm, no extension beyond capsule
3	<2.0 cm WITH extension beyond capsule
4	>=2.0 cm
5	Fixed/matted ipsilateral axillary nodes.
6	Axillary/regiona1 lymph nodes, NOS. Lymph nodes, NOS.
7	Internal mammary node(s), ipsilateral.
8	DISTANT Lymph Nodes: Cervical, NOS. Contralateral/bileteral axillary and/or internal mammary. Supraclavicular (transverse cervical). Other than above.
9	UNKNOWN; not stated.

Prior to 1998, the definition of "First Course" for all malignancies except leukemias was (*30*): All cancer-directed treatment administered to the patient within four months after the initiation of therapy (e.g. within 4 months of excisional biopsy). All modalities of treatment were included regardless of sequence or the degree of completion of any component method. Exceptions were: 1. If it was documented that the planned first course of therapy continued beyond or began after four months of initiation, include all as first course. 2. Should there be a change of therapy due to apparent failure of the original planned and administered treatment or because of progression of the disease, the later therapy should be excluded from the first course and considered part of a second course of therapy. From 1998, the 4-months time definition was increased to 1 year.

The SEER program have been described as the gold standard for cancer registration in the United States (*25*). The SEER registries have a comprehensive quality assurance program, by case-finding audits, education and training of personnel (*31*). The completeness of case incidence ascertainment is 98% and follow-up is 95% (*25*). In addition, each year the SEER registries reabstract medical records for a sample of cases to evaluate the accuracy of each of the data elements collected from the records (*32*).

For surgery and radiotherapy, several studies have used the Medicare to verify agreement between SEER records and insurance claims. Medicare is the primary health insurer for 97% of the US population 65 years or older (*32*). A good agreement between the SEER and Medicare claims was shown for breast cancer inpatients who underwent cancer-directed surgery (*33,34*). The accuracy of records was poorer for outpatients or for those who did not underwent cancer-directed surgery (no surgery or biopsy-only cases). The kappa measure of agreement (range from 0, no agreement, to 1, perfect agreement) was 0.70 for outpatients, 0.88 for inpatients (*34*).

For receipt of radiotherapy, Du et al found that more than 18% of Medicare patients identified as receiving radiotherapy were not so identified by SEER, and 7% of those identified by SEER were not identified by Medicare (*35*). The agreement was good for local and regional stages, with kappa respectively 0.82 and 0.77, whereas the agreement was poor for distant stage, kappa 0.074, and unstaged patients, kappa 0.50 (*35*). Virnig et al found a good agreement between SEER and Medicare with kappa 0.87 (*36*). The agreement was consistent across registries and over time during the study period 1991-1996 (*36*).

Limitations of the SEER data are the lack of informatin on comorbidity, on use of of diagnostic procedures, on recurrences. Receipt of systemic therapy or details of radiotherapy are not available.

In our studies using the SEER data, we have selected patients in whom breast cancer was the first primary tumour, histologically confirmed, hospital based, in whom cancer-directed surgery had been performed. These criteria correspond to cases where the SEER data have been shown to be the most reliable. Selection of patients was further limited by period of diagnosis, from 1988 (availability of detailed tumour extension data) to 1997 (before the change of treatment definition). The 9-registries

data was used as the main investigation database, reserving the other registries for future validation studies.

Section 3. AZ-VUB Breast Cancer data and selection

1. Description

In our hospital, the radiotherapy started functioning in 1984. The list of patients who were referred for radiation treatment at our hospital were recorded in a stand-alone dBase file. Maintenance was done by the chief nurse M.S. The structure of field records changed progressively over the years (Table 4). Data were related to billing and work management. Tumour data were "diagnose" and "TNM" (for definition of TNM, see Appendix).

Table 4. AZ-VUB dBase structure 1984-1995

1984	1985	1986	1987	1988	1989	1990-1991	1992-1995
DOSSIERNR	DOSSIERNR	JAAR	MAAND	JAAR	JAAR	JAAR	JAAR
NAAM	NAAM	MAAND	NAAM	MAAND	MAAND	MAAND	MAAND
VOORNAAM	VOORNAAM	NAAM	DOSSIERNR	NAAM	NAAM	NAAM	NAAM
DIAGNOSE	DIAGNOSE	DOSSIERNR	AMBULANT	DOSSIERNR	DOSSIERNR	DOSSIERNR	DOSSIERNR
TNM	TNM	AMBULANT	PATIENTNR	AMBULANT	PATIENTNR	PATIENTNR	PATIENTNR
JAAR	JAAR	PATIENTNR	SIMULATIE	PATIENTNR	EXTERN	EXTERN	EXTERN
MAAND	MAAND	SIMULATIE	HERSIMUL	SIMULATIE	VERW_ARTS	VERW_ARTS	VERW_ARTS
PATIENTNR	PATIENTNR	HERSIMUL	PLANNING	HERSIMUL	POSTNR	POSTNR	POSTNR
SELNR	EXTERN	PLANNING	BLOKKEN	PLANNING	AMBULANT	AMBULANT	AMBULANT
	POSTNR	BLOKKEN	MASKER	BLOKKEN	DIAGNOSE	DIAGNOSE	DIAGNOSE
	AMBULANT	MASKER	PLANNINGCT	MASKER	TNM	TNM	TNM
	SIMULATIE	PLANNINGCT	DIAGNOSE	PLANNINGCT	META	META	META
	HERSIMUL	DIAGNOSE	AANTALZITT	DIAGNOSE	SIMULATIE	SIMULATIE	SIMULATIE
	PLANNING	AANTALZITT	AANTALVELD	AANTALZITT	HERSIMUL	HERSIMUL	HERSIMUL
	BLOKKEN	AANTALVELD	TNM	AANTALVELD	PLANNING	PLANNING	PLANNING
	MASKER	TNM	TOT_DOSIS	TNM	BLOKKEN	BLOKKEN	BLOKKEN
	PLANNINGCT	TOT_DOSIS	TOESTELTYP	TOT_DOSIS	MASKER	MASKER	MASKER
	AANTALZITT	TOESTELTYP	EXTERN	TOESTELTYP	PLANNINGCT	PLANNINGCT	PLANNINGCT
	AANTALVELD	EXTERN	JAAR	EXTERN	AANTALZITT	AANTALZITT	AANTALZITT
	TOESTELTYP	POSTNR	BID	POSTNR	AANTALVELD	AANTALVELD	AANTALVELD
	BID	BID	TOESTELT2	HOSPITALIZ	TOESTELTYP	TOESTELTYP	TOESTELTYP
	TOESTELT2	TOESTELT2	AANTZITT2	GEHOSPIT	HOSPITALIZ	BID	BID
	AANTZITT2	AANTVELD2	AANTVELD2	FRACAMB	LIGDAGEN	TOESTELT2	TOESTELT2
	AANTVELD2	AANTZITT2	TARIF1	FRACHOSP	TOESTELT2	AANTZITT2	AANTZITT2
	TARIF1	TARIF1	TARIF2	BID	AANTZITT2	AANTVELD2	AANTVELD2
	TARIF2	TARIF2	TARIF3	TOESTELT2	AANTVELD2	TARIF1	TARIF1
	TARIF3	TARIF3	D1	AANTZITT2	TARIF1	TARIF2	TARIF2
	D1	D1	D2	AANTVELD2	TARIF2	TARIF3	TARIF3
	D3	D2	D3	TARIF1	TARIF3	D1	D1
	D2	D3	HUISARTS	TARIF2	D1	D2	D2
	HUISARTS	HUISARTS	ADRES	TARIF3	D2	D3	D3
	ADRES	ADRES	TEL	D1	D3	HUISARTS	HUISARTS
	TEL	TEL	HADRES	D2	STAD	ADRES	ADRES
	HTEL	HADRES	HTEL	D3	HUISARTS	TEL	TEL
	HADRES	HTEL	HPOSTNR	HUISARTS	TEL	HADRES	HADRES
	HPOSTNR	HPOSTNR	KONS	ADRES	HADRES	HTEL	HTEL
	KONS	KONS	OVERL	HTEL	HTEL	HPOSTNR	HPOSTNR
	OVERL	OVERL	STAD	HPOSTNR	HPOSTNR	KONS	KONS
	STAD	STAD	META	KONS	KONS	OVERL	OVERL
	VERW_ARTS	VERW_ARTS	VERW_ARTS	OVERL	OVERL	STAD	STAD
	META	META	SELNR	STAD	SELNR	SELNR	SELNR
	SELNR	SELNR		VERW_ARTS			TYPERT1
				SELNR			TYPERT2
							TYPERT3
							TYPERT4
							TYPERT5
							STANDARD

From 1994–1995, the listing of patient treatment became part of the hospital network appointment-scheduling-billing system. Clinical tumour data for breast cancer patients were collected in a separate database by Dr. C.C. The goal of the Breast Cancer database was to form the basis for evaluation of treatments outcomes. Source of data were paper and electronic medical records, radiation treatment files, and surgical-senology database maintained by Dr. J.L.

Current maintenance is by Dr. M.V. The maintained database structure is shown in Appendix A1 "Mamma juni 2005". The database is formed by the concatenation of the original dBase and the surgical database mentioned above, expanded with other descriptive fields. New records are appended whenever a patient receives a first simulation procedure (appointment for this first technical contact is determined with the hospital's appointment system). Data is abstracted from the radiation treatment folder which is completed at simulation time (Appendix A2). The radiation treatment folder itself is a summary of main pathology and treatment features. Subsequent data about the patient are added during the course of consultations and hospitalisations, outcome information communicated by colleagues, patient, family or social department. The current database is implemented in Filemaker. There is no direct link with the hospital's Electronic Medical Datafile (EMD).

2. Selection of patients

For the purpose of the present study, we aimed at a selection of patients that should be compatible with prior SEER studies. The criteria were:

- women
- first primary
- invasive carcinoma (exclude non epithelial tumors, sarcoma, lymphoma, in situ)
- unilateral (exclude synchronous bilateral invasive carcinomas; allow synchronous contralateral in-situ)
- pT1-2, (maximum tumour diameter <= 50 mm)
- M0 (non metastatic)
- Total number of examined axillary lymph nodes (ntot) known
- Total number of positive axillary lymph nodes (npos) known
- if pTx, use cT
- post-surgery radiation treatment delivered, within 4 months of definitive surgery or within 12 months if adjuvant chemotherapy and radiation was pre-planned in sequence with chemotherapy.
- exclude patients treated with Halsted operation
- exclude patients not operated or receiving biopsy only
- Further restrictions: ntot <=50, age 25-95 (extreme outliers).

3. Variables and endpoints of interest

Variables that were a priori considered for analyses were:

- hospital, intern or extern referral
- year of diagnosis
- age
- tumor size
- npos
- ntot
- quadrant
- laterality
- histology (for definitions, see Appendix)
- Estrogen Receptor status
- Progesterone receptor status
- neu-oncogen status
- histopathological grade (for definitions, see Appendix)
- type of surgery
- radiation details:
    - dosis
    - boost
    - sc
    - parasternal
    - duration RT
- chemotherapy, pre- and/or post-operative
- hormonotherapy, pre- and/or post-operative

Endpoints of interest were:

- local recurrence
- regional recurrence
- metastasis
- death from any cause
- death specifically from breast cancer

Source of the variables and and their coding are indicated below in Table 5 (Initial patient-tumour characteristics), Table 6 (Treatments), Table 7 (Outcomes), and Table 8 (Intermediary fields for translation). Imputation was done for partly missing tumour size and date of diagnosis.

Table 5. Initial patient-tumour characteristics

Label	Coding	Description	Source field	Source value
azvub	1=AZ-VUB<br>0=else or unknown	referring of patient	Verw. ziekenhuis | (JL_Chirurgen | JL_Adres HA | JL_Anapat)	"AZVUB";<br>not empty
YearDgc	continuous, year	year of diagnosis		calc: from earliest date first surgery or biopsy
AgeDgc	continuous, year	age at diagnosis		calc: from birth date and date diagnosis
left	1=left breast<br>0=right or unknown	side	LRi	1<- "L"
innerkwd	1=medial quadrant<br>0=else		Quadrant_i	1<- "SI" or "II"
histduct	1=ductal carcinoma<br>0=else		Anapat_i<br>Anapat_i_2	0<- not(IDC or IDC+is or IDC+is+LCIS) in any field
er	0=ER negative<br>1=weak positive<br>2=moderate<br>3=strong positive<br>9=unknown		OestrogeenRec<br>JL_ERICA	1<- "-"
pr	0=PR negative<br>1=weak positive<br>2=moderate<br>3=strong positive<br>9=unknown		ProgesteronRec<br>JL_PROG	1<- "-"
erval	continuous (U)		OestrogeenRecWaarde	as is
prval	continuous (U)		ProgesteronRecWaarde	as is
neu2	1=positive<br>0=negative or weak+<br>9=unknown		Neu<br>JL_Neu_Onco	1<- pos
g34	1=high grade (poorly- or undifferentiated)<br>0=else		AZ-graad<br>DiffG_BSR_i<br>DiffG_WHO_i<br>JL_G	1<- >2
EodSize	continuous, mm	max diameter of tumor	tumor diameter (cm)<br>JL_tumordiameter<br>JL_Macrosc_Diam<br>Ti<br>pTi	check consistency (units and T-stage)
npos	continuous, n	number of involved axil. lymph nodes	NTotPos, N1..4Pos,<br>JL_#LymfeNN_positief,<br>JL_Apex, JL_NivI..III	count
ntot	continuous, n	number of examined axil. lymph nodes	NTotTot, N1..4Tot,<br>JL_opapex,<br>JL_opi..iii	count

Table 6. Treatments

Label	Coding	Description	Source field	Source value
bcs	1=breast conserving surgery<br>0=else		HKi_def	1<- "Quadrantectomie", "Tumorectomie", "Tumor/quad. ect.", "Partiele mastectomie"
chem	1=adjuvant chemotherapy<br>0=no or unknown	post-operative chemo, with or without pre-op chemo	Chemo	1<- "Postop" or "postop"
horm	1=adjuvant hormonotherapy<br>0=no or unknown	post-op hormono-therapy, with or without pre-op horm.	Hormonotherapie	1<- "Postop" or "postop" or ("Post-chemo" and chem=1)
neochem	1=pre-operative chemotherapy<br>0=no or unknown	pre-op chemo, with or without post-op chemo	Chemo	1<- "Preop"
neohorm	1=pre-operative hormonotherapy<br>0=no or unknown	pre-op hormono-therapy, with or without post-op horm.	Hormonotherapie	1<- "Preop"
rt	1=adjuvant RT<br>0=no RT<br>9=unknown or not adjuvant		RTEnergie_i	0<- "Geen"<br>9<- empty
Rtdose	continuous (Gy)	RT dose delivered	Dosis_i	as is (correction decimal separator "," ".")
Rtboost	continuous (Gy)	RT boost dose	BoostDosis	as is (correction decimal separator "," ".")
Rtsc	1=SC irradiated<br>0=no<br>9=unknown	RT supra-clavicular,axillary	SCDosis	1<- >0
Rtimc	1=IMC irradiated<br>0=no<br>9=unknown	RT internal mammary chain	ParasternaleRX	1<- "Y"
Rtduur	continuous (days)	duration RT	Duur_i	as is

Table 7. Outcomes

Label	Coding	Description	Source field	Source value
LRR	1=local and/or regional recurrence occurred<br>0=no	flag	Lokaal recidief i	1<- not ("Geen" or empty)
Meta	1=metastasis occurred<br>0=no	flag	Metas	
Sec	1=second cancer occurred<br>0=no	flag	TweedeTumor	1<- "Na borst"
Died	1=died any cause<br>0=alive at last follow-up	flag for overall survival	Died	as is
RorD	1=(LRR=1 or Meta=1 or Died=1)<br>0=alive without recurrence or metastasis	flag for DFS		calc: 1<- (LRR=1 or Meta=1 or Died=1)
RMSorD	1=(LRR=1 or Meta=1 or Died=1 or Sec=1)<br>0=alive without any cancer	flag for cancer-free survival		calc: 1<- (LRR=1 or Meta=1 or Died=1 or Sec=1)
Dod	1=died of breast cancer<br>0=alive or died from other cause	flag for BC specific survival	OorzaakOverlijden	1<- Died=1 and (LRR=1 or Meta=1)
t2LRR	time to local recurrence<br>(equals t2Lfu if LRR=0)	continuous (months)	DatumRecidief_i	calc
t2Meta	time to metastasis<br>(equals t2Lfu if Meta=0)	continuous (months)	DatumMetas	calc
t2Sec	time to second cancer	continuous (months)		calc
t2Dod	time to death of breast cancer<br>(equals t2Lfu if Dod=0)	continuous (months)		calc
t2RorD	time to recurrence, metastasis or death<br>(equals t2Lfu if RorD=0)	continuous (months)		calc
t2RMSorD	time to RMSorD	continuous (months)		calc
t2Lfu	time to death or last follow-up	continuous (months)		calc

Table 8. Intermediary fields

Field	Coding	Remark
seqnum	0=first primary<br>1=second primary<br>2=third primary...<br>98=99th primary<br>99=duplicate	0 means no previous cancer;selection excludes all cases seqnum > 0
sex	0=male<br>1=female<br>9=unknown	
Laterality	1=right<br>2=left<br>3=one side, R or L unspecified<br>4=bilateral<br>9=no information	
grade	1-4=well, moderately, poorly, undifferentiated<br>9=unknown	converted from AZ-graad, DiffG_BSR_i, DiffG_WHO_i, JL_G
DateDgc		192 cases unknown.
Laatste_Datum	Last date for which some status of patient is known; if no date recorded: 1/1/1111	
t2Lfu		(Laatste_Datum-DateDgc)/30.5
SeerReg	assigns dummy registry number, when later we merge with the SEER database	
surg	0=mastectomy<br>1=breast conserving<br>7=no cancer surgery w/wo biopsy<br>8=biopsy, debulking only, other procedure<br>9=unknown	

4. Imputation of partly missing tumour size

It is based on the distribution of known tumor size by pT-stage, taking into account the distribution skewness and rounding of measurements, using Table 9.

Table 9. Distribution of tumour sizes by pT-stage

pT-stage	Mean tumor size (mm)	Median tumor size (mm)	Max range for random "jitter" (mm)
pT1a	4.1	5	3-5
pT1b	8.6	9	6-10
pT1c	15.9	15	11-20
pT1	13.8	15	3-20
pT2	30.4	30	21-50
pT3	70.1	65	51-100

The imputation procedure is as follows.

1) Case clinical-radiological size available:

If clinical size matches pT, use clinical size.

If mismatch and not T4, interpolate betwee pT mean size (see Table) and clinical size. If interpolation larger than pT, use largest pT boundary value. Example: pT1 (size <=20 mm), clinical size 50 mm, the value half-way between mean 13.8 mm and the recorded clinical size is 31.9, which is in contradiction with pT1 -> use 20 mm. If pT1 and recorded clinical size 25 mm, value half-way is 19.4 mm -> use 19.4 mm.

2) Case clinical size not available:

Use median size according to pT1-pT3, with random "jitter" (Table).

If pT0, assign size as 0.1 mm.

If pT not available, or pTis or pT4: use cT0-cT3 if available.

3) Else, record as missing.

Note that patients with tumour size >50 mm are not analyzed in the present paper.

The validity of the imputation for tumour size was subsequently verified in patients for whom pathological description was found (Table 10). The average signed error was 1 mm, and the average absolute error was 5 mm.

Table 10. Check of validity of tumour size imputation

imputed size (mm)	From AnaPath	Error (mm)	abs(Error)	AnapatExtra_i
5	5	0	0	0.5 cm, invasief mucoid ca.
9	10	-1	1	1 cm, beginnende invasie, plaatselijk tubulair
9	9	0	0	0.9 cm
9	5	4	4	0.5 cm, marge0.3 cm, gr1
9	11	-2	2	1.1 cm
9	10	-1	1	1 cm,
12	7.8	4	4	< 1 cm
12	12.5	0	0	>1 cm,
13	12	1	1	1.2 cm,
13	3.5	9	9	Matig tot weinig gedifferentieerd duktaal ca, <0.5 cm, gr3
13	18	-5	5	1.8 cm, 0.5 cm vanaf diepe snijvlak; DCIS: beperkt
13	16	-3	3	1.6 cm, M. Reclus
13	12	1	1	1.2 cm, perineurale uitbreiding, + Pagetoide intraductale sprei- ding
14	12	2	2	1.2 cm; DCIS-->comedotype
14	10	4	4	1 cm;<br>top: 0/1
14	20	-6	6	2 cm
14	20	-6	6	2 cm, gr1
14	19	-5	5	1.9 cm
14	20	-6	6	2 cm; EDCIS
15	18	-3	3	1.8 cm, gr. 2
15	17	-2	2	1.7 cm
15	20	-5	5	2 cm, matig gedifferentieerd, gr2-3, klein nestje residueel tumorweefsel,<br>perineurale invasie
15	12	3	3	1.2 cm
15	15	0	0	1.5 cm
15	9	6	6	0.9 cm,
16	10	6	6	1 cm; vermoedelijk lymfvatinvasie
16	20	-4	4	2 +0.5 cm, enkele haardjes DCIS comedotype, M. Reclus
16	9	7	7	0.9 cm, 1 cm marge, clips !
16	5	11	11	0.5 cm, M. Reclus
16	17	-1	1	1.7 cm, marge > 1 cm, E&E
16	15	1	1	1.5 cm
16	10	6	6	1 cm, wrschl. apocrien
16	20	-4	4	2 cm
16	7.8	9	9	<1 cm, DCIS tot ver buiten de tumorhaard, M. Reclus
16	20	-4	4	tubulair ca. met multifocale distributie binnen een nodule van 2 cm
16	9	7	7	0.9 cm
16	9	7	7	0.9 cm, tubulocribriform, invasief carcinoom;<br>DCIS (kleincellig, weinig ontw.)
17	15	2	2	1.5 cm, scirrheus carcinoom
17	20	-3	3	2 cm, M. Reclus
17	17	0	0	1.7 cm
17	19	-2	2	1.9 cm, M. Reclus in regressie
17	18	-1	1	1.8 cm
18	12	6	6	1.2 cm; M. Reclus
18	17	1	1	1.7 cm + 0.5 cmgr2-3, bifocaal, bloedvatinvasie

18	18	0	0	1.8 cm
18	16	2	2	1.6 cm
18	3.5	14	14	<0.5 cm, gr3
18	13	5	5	1-3 cm
18	20	-2	2	2 cm; + comedotype
20	18	2	2	1.8 cm,
24	45	-21	21	multicentrisch comedo c.i.s., > 4 cm
24	20	4	4	2 cm + residu, ILC-variant (closely aggregated type)
24	22	2	2	2.2 cm
24	15	9	9	1.5 cm, tegen en in snijvlak
25	30	-5	5	3 cm,
25	25	0	0	2.5 cm, spierinvasie (invasie fascie + oppervl. deel M. pectoralis pectorale vet + wand v/d fasciale venen)
25	28	-3	3	2.8 cm, diep
26	25	1	1	2.5 cm, lymfvatinvasie
26	23	3	3	2-3 cm
26	28	-2	2	2.8 cm
27	25	2	2	2.5 cm
27	30	-3	3	3 cm, type comedo,perineurale groei
27	21	6	6	2.1 cm
28	30	-2	2	3 cm + 2 cm, + comedocmponent
29	25	4	4	2.5 cm, totop 0.1 cm van het diepe snijvlak, atypische variant van medullair carcinoom
29	50	-21	21	bifocaal, 5 cm + 3.5 cm
30	35	-5	5	3.5 cm, type colloid, totaan thoracale snijvlak
31	35	-4	4	3.5 cm, solied
31	35	-4	4	3.5 cm
32	24	8	8	2.4 cm
33	23	10	10	2.3 cm
33	25	8	8	2.5 cm; M. Reclus
33	36	-3	3	3.6 cm
35	40	-5	5	4 x 3.5 cm
36	21	15	15	2.1 cm, comedotype, vaatpermeatie
36	22	14	14	2.2 cm
36	45	-9	9	4.5 cm
37	17.5	20	20	>1.5 cm, scirrheus carcinoom; M. Reclus
37	35	2	2	3.5 cm, lymfogene invasie van de dermis; totin de pars papillaris van de dermis onder tepel; in de tepel zelf M. Paget
62	50	12	12	5 cm, type colloid carcinoom

5. Imputation of partly missing date of diagnosis

An attempt to impute date of first diagnosis was attempted when no explicit date was recorded.

1) if date start RT is known:

- case no preop chemo: DateDgc <- date Start RT minus 21 days

(average 3 weeks between surgery and RT).

- case preop chemo: DateDgc <- date Start RT minus (number of cycli + 1)*21 days

(average 3 weeks/cycli and assume 3 weeks between end chemo and RT).

2) if startRT is unknown, but date end RT is known:

- case total RT dose known: (=Rtdose+Rtboost) / 2 (usual fraction dose) = number of fraction.

Number of fraction / 5 (usual number of fractions per week), multiply by 7 -> estimated duration of RT in days.

DateDgc <- date end RT minus (estimated duration RT + average delay 21 days).

- case total RT dose unknown:

DateDgc <- date end RT minus 61 days (average duration RT 40 days + average delay 21 days).

3) else, neither start RT or end RT known:

DateDgc <- 3/3/3333 (we assign dummy date of origin beyond next millenium, consequently time from dummy origin to any event during our lifetime will be negative).

6. Database difficulties

We will briefly mention difficulties in exploring our own database. The two major problems (most time consuming) were related to dates, and to the sequence of primaries.

Discrepancies in dates of death were encountered. We noted a high frequency of first day of the month, and a high frequency of 15th day. These probably indicated rounding error when the exact date of death was unknown. Whenever there was a discrepancy between the database and the medical record, the shorter survival duration was selected. Rarely there was a mismatch between date of death mentioned in the medical record, and the date of death mentioned in the autopsy report. Whenever available, we used the autopsy date. Some typing errors were noted, e.g. month larger than 12, o or O for 0. Some difficulties were related to century date rollover due to the use of 2-digits year number.

The other problem was the sequence of primaries. Sometimes this could only be ascertained by browsing through several medical reports.

7. Final selection

Records for whom ntot, npos, tumour size and diagnostic dates were entirely missing were rejected.

After cleaning records, verification and correction of data using the medical and the radiation treatment files, there were 3907 records identified. Selection of the data used a Filemaker script and retrieved 2109 records. After exclusion of ntot outliers (1 case) and age outlier (1 case 21 years old), and exclusion of cases diagnosed before 1984 (the radiotherapy department had not yet been created), there were 2092 individual records available for analysis. All these patients received post-surgery radiation treatment, not more than 4 months after surgery (without adjuvant chemotherapy), or not more than 1 year after surgery (if adjuvant chemotherapy was given prior to radiation treatment).

Section 4. Methods

The outcome of interest in this paper is the time to death (event), or survival time (*37*). Details of the procedures are available in textbooks. They are reproduced here for convenience.

The survival probability $S(t)$ is the probability that a patient survives from the time origin to a specified time t. Since not all patients can be observed during their whole lifetime, censoring occurs, that is, patients are still alive (no event) at the end of the observation period, or are lost to follow-up before the end of the observation period.

Estimation of survival used the Kaplan-Meier product-limit method (*38*). Denoting j an index for the ordered survival times (t_1, t_2, ... t_{j-1}, t_j), r_j the number of patients alive (at risk) just before t_j, and d_j the number of events at t_j, the estimated survival probability is

$$S(t_j) = S(t_{j-1})(1 - d_j/r_j)$$

where t_0=0 and $S(0) = 1$.

Equivalently

$$S(t) = (1 - d_1/r_1)(1 - d_2/r_2) \ldots (1 - d_{j-1}/r_{j-1})(1 - d_j/r_j)$$

where $t_j \leq t$.

Note that $S(t)$ is the product of successive conditional survival rates.

The Kaplan-Meier survival estimate assumes that censoring is non-informative.

A closely related function is the hazard rate $h(t)$, that is, the conditional failure rate of individuals who are under observation at time t having an event at that time. The hazard can be computed from the survival probability by

$$h(t) = -\mathrm{d}/\mathrm{d}t[\log S(t)]$$

where log denotes the natural logarithm.

The cumulative hazard $H(t)$ is defined as the integral of the hazard, and is related to the survival by

$$H(t) = -\log[S(t)].$$

The cumulative hazard can also be computed by the Nelson-Aalen estimator:

$$H(t) = d_1/r_1 + d_2/r_2 + \ldots + d_{j-1}/r_{j-1} + d_j/r_j$$

where $t_j \leq t$.

$H(t)$ is the sum of successive death rates.

Multivariate survival analyses used the Cox proportional hazards regression model (*39,40*). Denote covariates X_1, X_2, ... X_p (for example X_1=histology, X_2=grade, X_3=location, etc.), and X_{i1}, X_{i2}, ... X_{ip} as the covariates for the patient i, where i = 1, 2, ..., n. The Cox model specifies the hazard for the patient i as

$$h_i(t) = h_0(t) \exp(b_1X_{i1} + b_2X_{i2} + \ldots + b_pX_{ip}),$$

where $h_0(t)$ is an unspecified nonnegative function of time called the baseline hazard, and b_1, b_2, ... , b_p are coefficients.

Taking the natural logarithm and suppressing the subscripts i, it might be written as

$\log[h(t)] = \log[h_0(t)] + b_1X_1 + b_2X_2 + ... + b_pX_p$.

The unspecified function $h_0(t)$ is the hazard $h(t)$ when $X_1 = ... = X_p = 0$.

Estimates of the coefficients b_1, b_2, ... , b_p are obtained by the method of maximum partial likelihood.

The Cox model assumes that the ratio of the hazard functions for any two patient subgroups (i.e. two groups with different values of the explanatory variables X_1, X_2, ..., X_p) is constant over follow-up time.

In the model, the coefficient b_j (for the *j*th covariate) represents the increase in log hazard if the covariate X_j is increased by one unit and all other covariates are held constant. When X_j is continuous, this implies the assumption that the relationship between the covariate and the log hazard, i.e. the functional form, is linear.

Several types of residuals (differences between expected and observed survival) are defined in the Cox model. The paper used the Schoenfeld residuals to assess the porportional hazards assumption or constancy in time of the hazard ratios. The martingale residuals and the generalized additive model (GAM) was used to assess the functional form of continuous covariates (*40*).

Statistical computations used JMP (SAS Institute Inc, Cary, NC, USA) and Splus (Insightful Corp, Seattle, WA, USA).

Section 5. Concordance SEER and AZ-VUB

Previous research work on the SEER data have shown that registry geographical area, age at diagnosis, tumour size, extent of nodal involvement, tumour location, histology, histopathological grade, hormone receptor status, surgery, radiation, were statistically significant prognostic factors, while laterality was not significant (*13*).

For the AZ-VUB, Table 11 based on the 2092 available records shows results of multivariate models. As for subsequent Tables, hazard ratio >1 indicates relative increased risk of death vs. reference for categorical variables, or relative increase per unit change for continuous variables. Tumour location, histology, estrogen receptor and grade appeared non-significant. Nevertheless, the values of the hazard ratios are concordant with the SEER. We note that patients internally referred appeared to have a better prognosis. This suggests the possibility of geographical variation, but this was not investigated.

Table 11. AZ-VUB, overall mortality, all cases.

Variable	hazard ratio	p
Internal referral (vs. external)	0.738	0.009
tumour left side (vs. right)	1.055	0.600
tumor medial location (vs. else)	1.135	0.300
histology ductal (vs. else)	1.291	0.077
Estrogen Receptor negative (vs. else)	1.288	0.110
Progesterone rec. negative (vs. else)	1.423	0.019
Grade 3-4 (vs. else)	1.227	0.066
Breast conserving surgery (vs. mastectomy)	0.775	0.046
Year of diagnosis (continuous, year)	0.952	0.000
Age at diagnosis (continuous, year)	1.034	0.000
Tumour size (continuous, mm)	1.019	0.001
Number of positive nodes (continuous, n)	1.100	0.000
Number of examined nodes (continuous, n)	0.963	0.000

Rsquare=0.109, Likelihood ratio test=242.

Figure 1. Estimated relationship between age at diagnosis (AgeDgc, years), tumour size (EodSize, mm), number of positive nodes (npos, n), and number of examined nodes (ntot, n) with the risk of death. Dashed curves: 2 standard errors.

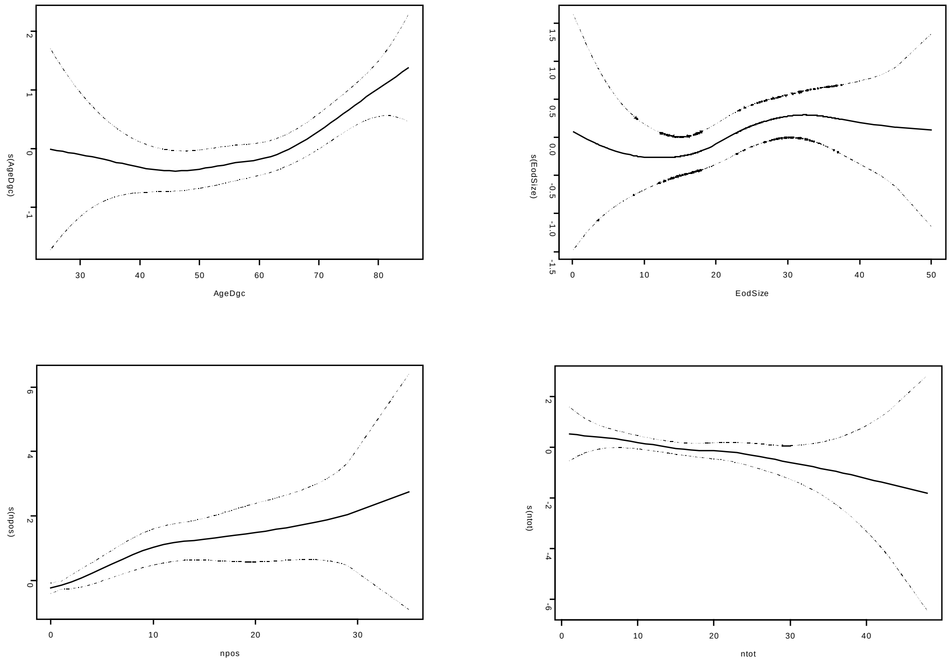

The functional forms for age, tumour size and number of positive nodes from the AZ are non-linear (Figure 1). The shapes are in keeping with earlier SEER studies (*16*,*21*,*22*). Using the respective transforms found from the SEER data,

- age + $|\text{age}-47.5|^{1.5}$ for age (in years),
- exp(-exp(-(size-15)/10)) for size (in mm),
- $\log_e$((npos+0.5)/(ntot-npos+0.5)), for nodes numbers,

we note that the global model was slightly improved (larger Rsquare and likelihood ratio tests, Table 12), and the transforms satisfied the linearity test (Table 13). Although deviation from the proportional hazards assumption was significant for histology and for the nodal transform (Table 12), the rho-values indicated small deviations, excluding thus major deviation from the proportional hazards.

Table 12. Model replacing continuous variables with non-linear transforms. Hazard ratios for all cause mortality and tests of propotional hazards assumption.

Variable	hazard ratio	p	Test proportional hazards rho	p
tumor medial location (vs. else)	1.161	0.220	0.062	0.220
histology ductal (vs. else)	1.206	0.190	-0.149	0.003
Estrogen Receptor negative (vs. else)	1.207	0.220	-0.098	0.055
Progesterone rec. negative (vs. else)	1.322	0.057	0.015	0.767
Grade 3-4 (vs. else)	1.244	0.051	-0.074	0.138
Breast conserving surgery (vs. mastectomy)	0.89	0.370	-0.029	0.562
Year of diagnosis (continuous, year)	0.952	0.000	-0.009	0.865
Age transform: age + $\lvert age-47.5\rvert^{1.5}$	1.007	0.000	0.042	0.385
Tumour size transform: exp(-exp(-(size-15)/10)), size in mm)	2.006	0.005	0.014	0.772
Log odds nodal involvement: $\log_e$((npos+0.5)/(ntot-npos+0.5))	1.311	0.000	-0.120	0.015

Rsquare=0.111, Likelihood ratio test=245.

Table 13. Non-linearity test based on GAM procedure. Untransformed variables significantly non-linear (P<0.05).

	untransformed		transformed	
	Chisq	P(Chi)	Chisq	P(Chi)
Age variable	24.50	0.00	5.23	0.16
Tumour size variable	9.63	0.02	6.67	0.08
Log odds nodal involvement			5.86	0.12
Number of positive nodes	7.98	0.05		
Number of examined nodes	2.50	0.47		
Year of diagnosis	1.30	0.73	1.79	0.62

We also examined disease-free survival (defined as survival without recurrence, metastasis or second primary) and recurrence-free survival (defined as survival without recurrence or metastasis). The models are qualitatively similar (Table 14).

Table 14. Models with disease-free and recurrence-free survival endpoints

Variable	disease-free hazard ratio	p	reccurence-free hazard ratio	p
tumor medial location (vs. else)	1.228	0.047	1.219	0.067
histology ductal (vs. else)	1.075	0.540	1.038	0.760
Estrogen Receptor negative (vs. else)	1.199	0.170	1.220	0.150
Progesterone rec. negative (vs. else)	1.266	0.065	1.291	0.050
Grade 3-4 (vs. else)	1.184	0.080	1.225	0.042
Breast conserving surgery (vs. mastectomy)	0.979	0.840	0.951	0.650
Year of diagnosis (continuous, year)	0.972	0.010	0.967	0.004
Age transform	1.004	0.000	1.005	0.000
Tumour size transform	1.967	0.001	2.145	0.000
Log odds nodal involvement	1.269	0.000	1.301	0.000

We conclude from this section that:

1) The SEER data do not have information on local-regional or metastatic recurrence. Analysis of the AZ-VUB data indicates that overall-survival is an acceptable surrogate of specific disease outcome.

2) Results from modeling the SEER data are applicable to the AZ-VUB data, indicating the relative similarity of breast cancer in the two databases. This is important since this allows us to proceed to merging for direct comparisons.

Section 6. Comparison of SEER and AZ-VUB treatment outcome

The two databases were merged, 83686 patients from SEER, 2092 patients from AZ-VUB. The median follow-up for patients alive from the SEER was 73 months (mean 76, range 1-143), from the AZ-VUB was 70 months (mean 74, range 1-250).

Figure 2 shows the unadjusted overall survival comparison of the SEER and AZ-VUB patients, regardless of treatment and nodal status. There is a statistically significant overall survival advantage for the AZ-VUB patients. Table 15 shows the respective 5-year and 10-year overall survival, indicating 1% survival advantage for AZ-VUB patients at 5-year and 3% at 10-year.

Figure 2. Unadjusted overall survival

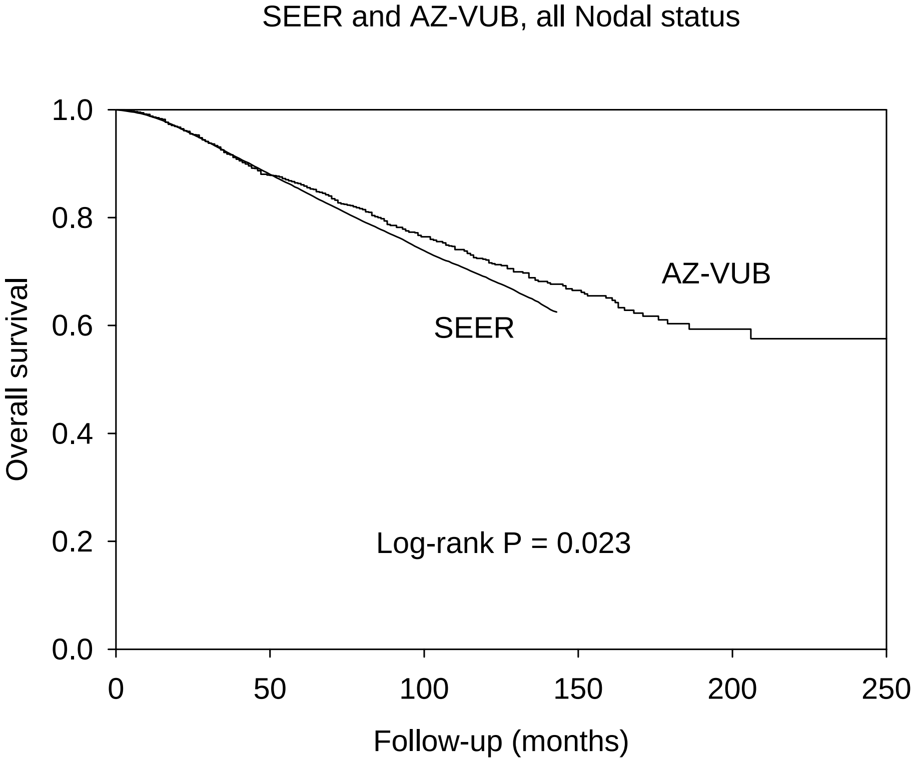

Table 15. Unadjusted overall survival estimates

	SEER	AZ-VUB
5-year OS	85.1% (0.13%)	86.1% (0.89%)
10 year OS	69.0% (0.24%)	72.1% (1.37%

The question that arises: is the survival difference attributable to differences in tumour and/or treatment characteristics, or to differences in the populations unrelated to tumour/treatment? The multivariate model from Table 16 indicates that the survival advantage of AZ-VUB patients remains significant when adjusted with other characteristics. But, for recall, all AZ-VUB patients received RT, therefore the model cannot answer whether the survival advantage is due to treatment or to population differences. Thus, more detailed analyses are required. Considering that there is an overall survival difference both in the above unadjusted and in multivariate analyses, it appeared justified to perform subgroup comparisons.

Table 16. Overall mortality, all cases: reduced mortality hazard ratio 0.819 for AZ-VUB patients vs. SEER.

Variable	hazard ratio	p
tumor medial location (vs. else)	1.120	0.000
histology ductal (vs. else)	1.132	0.000
Estrogen Receptor negative (vs. else)	1.465	0.000
Progesterone rec. negative (vs. else)	1.172	0.000
Grade 3-4 (vs. else)	1.249	0.000
Breast conserving surgery BCS (vs. mastectomy)	1.061	0.110
RT (vs. no RT)	0.944	0.064
AZ (vs. SEER)	0.819	0.000
Year of diagnosis (continuous, year)	0.968	0.000
Age transform	1.007	0.000
Tumour size transform	2.850	0.000
Log odds nodal involvement	1.293	0.000
interaction BCSxRT	0.772	0.000

Before proceeding to subgroup comparisons, we need to examine the distribution of treatments (Table 17), in order to determine what comparisons can be reasonably considered.

Table 17. Distribution of surgical (bcs) and radiation (rt) treatments by registry (isaz), by nodal status (N1) and extent of nodal involvement (n4). "mbb"= might be biased.

isaz 0=SEER 1=AZ-VUB	N1 0=node-negative 1=node-positive	n4 0= less than 4 pos.nodes 1= 4+ pos.nodes	bcs 0=mastectomy 1=breast conserving	rt 0=no RT 1=RT delivered	number of cases	remark	row number
0	0	0	0	0	31717		1
0	0	0	0	1	1021	mbb	2
0	0	0	1	0	2941	mbb	3
0	0	0	1	1	22391		4
0	1	0	0	0	10225		5
0	1	0	0	1	945	mbb	6
0	1	0	1	0	999	mbb	7
0	1	0	1	1	4589		8
0	1	1	0	0	4856		9
0	1	1	0	1	1996		10
0	1	1	1	0	491		11
0	1	1	1	1	1515		12
1	0	0	0	1	510		13
1	0	0	1	1	609		14
1	1	0	0	1	414		15
1	1	0	1	1	198		16
1	1	1	0	1	281		17
1	1	1	1	1	80		18

Table 17 shows that among the SEER patients, node-negative or node-positive with less than 4 positive nodes mastectomy patients receiving radiation (RT) is uncommon (row 2 vs. 1, and row 6 vs. 5). Omission of RT after breast conserving surgery, although more substantial, is also uncommon (row 3 vs. 4, row 7 vs. 8, row 11 vs. 12). The distribution is in keeping with general guidelines (*4*). Comparisons among the SEER patients have been done before (*8,14*). To repeat the same analyses will neither provide additional information nor solve the problem of these treatment biases. However, among the AZ-VUB patients, per definition all patients received RT. Therefore the best comparisons that might be performed is between the least likely biased treatments from the SEER and the AZ-VUB.

The comparisons are done within the context of multivariate models in Tables 18–22. Labels abbreviations refer to Table 17. In each of the Tables 18–22, results of interest are highlighted in italics.

a) Breast conserving surgery patients who received radiation: modeling indicates no significant difference in survival outcome between SEER and AZ-VUB patients treated with BCS and RT (Table 18).

Table 18. Breast conserving surgery with radiation (bcs=1 and rt=1), all nodal status. NA=not applicable. AZ vs. SEER not significant.

Variable	hazard ratio	p
tumor medial location (vs. else)	1.136	0.006
histology ductal (vs. else)	1.158	0.001
Estrogen Receptor negative (vs. else)	1.441	0.000
Progesterone rec. negative (vs. else)	1.280	0.000
Grade 3-4 (vs. else)	1.349	0.000
Breast conserving surgery BCS (vs. mastectomy)	NA	NA
RT (vs. no RT)	NA	NA
AZ (vs. SEER)	*0.864*	*0.160*
Year of diagnosis (continuous, year)	0.964	0.000
Age transform	1.007	0.000
Tumour size transform	3.297	0.000
Log odds nodal involvement	1.349	0.000
interaction BCSxRT	NA	NA

b) Mastectomy patients: RT did not appear significant, but a survival advantage was noted for AZ-VUB patients (Table 19).

Table 19. Mastectomy patients (bcs=0) with or without radiation, all nodal status. NA=not applicable. RT not significant, but AZ significantly reduced mortality.

Variable	hazard ratio	p
tumor medial location (vs. else)	1.107	0.000
histology ductal (vs. else)	1.124	0.000
Estrogen Receptor negative (vs. else)	1.492	0.000
Progesterone rec. negative (vs. else)	1.144	0.000
Grade 3-4 (vs. else)	1.210	0.000
Breast conserving surgery BCS (vs. mastectomy)	NA	NA
RT (vs. no RT)	*0.976*	*0.450*
AZ (vs. SEER)	*0.779*	*0.000*
Year of diagnosis (continuous, year)	0.967	0.000
Age transform	1.007	0.000
Tumour size transform	2.731	0.000
Log odds nodal involvement	1.280	0.000
interaction BCSxRT	NA	NA

c) Node-negative mastectomy patients: comparing AZ-VUB patients who received RT with SEER patients who did not receive RT shows no significant difference for RT (Table 20).

Table 20. Node negative mastectomy (N1=0, bcs=0), AZ (rt=1) vs. SEER (rt=0).

Variable	hazard ratio	p
tumor medial location (vs. else)	1.096	0.006
histology ductal (vs. else)	1.113	0.000
Estrogen Receptor negative (vs. else)	1.309	0.000
Progesterone rec. negative (vs. else)	1.084	0.053
Grade 3-4 (vs. else)	1.135	0.000
Breast conserving surgery BCS (vs. mastectomy)	NA	NA
RT (vs. no RT)	*0.954*	*0.640*
AZ (vs. SEER)	NA	NA
Year of diagnosis (continuous, year)	0.985	0.010
Age transform	1.008	0.000
Tumour size transform	2.787	0.000
Log odds nodal involvement	1.152	0.000
interaction BCSxRT	NA	NA

d) Node-positive mastectomy patients: comparing AZ-VUB patients who received RT with SEER patients who *did not receive* RT shows a significant advantage with RT (Table 21). The mortality reduction with RT is maintained regardless of few nodes involved (columns "1-3 positive nodes) or more nodes involved (columns "4+ positive nodes").

Table 21. Node-positive mastectomy (N1=1, bcs=0), AZ (rt=1) vs. SEER (rt=0).

	all nodal status		1-3 positive nodes		4+ positive nodes	
Variable	hazard ratio	p	hazard ratio	p	hazard ratio	p
tumor medial location (vs. else)	1.090	0.001	1.106	0.074	1.078	0.280
histology ductal (vs. else)	1.115	0.000	1.136	0.007	1.104	0.046
Estrogen Rec. negative (vs. else)	1.456	0.000	1.587	0.000	1.556	0.000
Progest. rec. negative (vs. else)	1.145	0.000	1.193	0.004	1.286	0.000
Grade 3-4 (vs. else)	1.211	0.000	1.297	0.000	1.300	0.000
Breast cons. surg. BCS (vs. mast.)	NA	NA	NA	NA	NA	NA
RT (vs. no RT)	*0.758*	*0.000*	*0.591*	*0.000*	*0.752*	*0.005*
AZ (vs. SEER)	NA	NA	NA	NA	NA	NA
Year of diagnosis (cont., year)	0.969	0.000	0.961	0.000	0.950	0.000
Age transform	1.007	0.000	1.006	0.000	1.004	0.000
Tumour size transform	2.796	0.000	2.998	0.000	2.408	0.000
Log odds nodal involvement	1.287	0.000	1.243	0.000	1.254	0.000
interaction BCSxRT	NA	NA	NA	NA	NA	NA

e) Node-positive mastectomy patients with 4+ positive nodes: comparing SEER with AZ-VUB patients who *both received* RT shows no significant difference between SEER and AZ-VUB patients (Table 22).

Table 22. 4+ positive nodes mastectomy (n4=1, bcs=0), AZ (rt=1) vs. SEER (rt=1).

Variable	hazard ratio	p
tumor medial location (vs. else)	1.265	0.025
histology ductal (vs. else)	1.096	0.270
Estrogen Receptor negative (vs. else)	1.367	0.003
Progesterone rec. negative (vs. else)	1.324	0.004
Grade 3-4 (vs. else)	1.173	0.028
Breast conserving surgery BCS (vs. mastectomy)	NA	NA
RT (vs. no RT)	NA	NA
AZ (vs. SEER)	*0.942*	*0.580*
Year of diagnosis (continuous, year)	0.945	0.000
Age transform	1.004	0.000
Tumour size transform	1.460	0.013
Log odds nodal involvement	1.246	0.000
interaction BCSxRT	NA	NA

We conclude from this section that, within multivariate models that adjust for tumour characteristics:

1) With the exception of node-negative mastectomy patients in whom the comparison appears inconclusive (Table 20),

2) the survival advantage of AZ-VUB vs. SEER was observed only whenever the comparison was with SEER patients who did not received RT (Table 21, all 3 columns),

3) whereas, whenever AZ-VUB and SEER patients both received RT, no significant survival difference was found (Table 19 and 22).

4) The small difference in overall survival between the overall SEER and AZ-VUB patients noted at the beginning of this Section (Figure 2) appears thus attributable to a large disadvantage in survival among node-positive mastectomy patients who did not receive RT as shown by the hazard ratios of Table 21, or graphically in Figure 3.

Figure 3. Unadjusted overall survival, 1-3 positive nodes mastectomy patients.

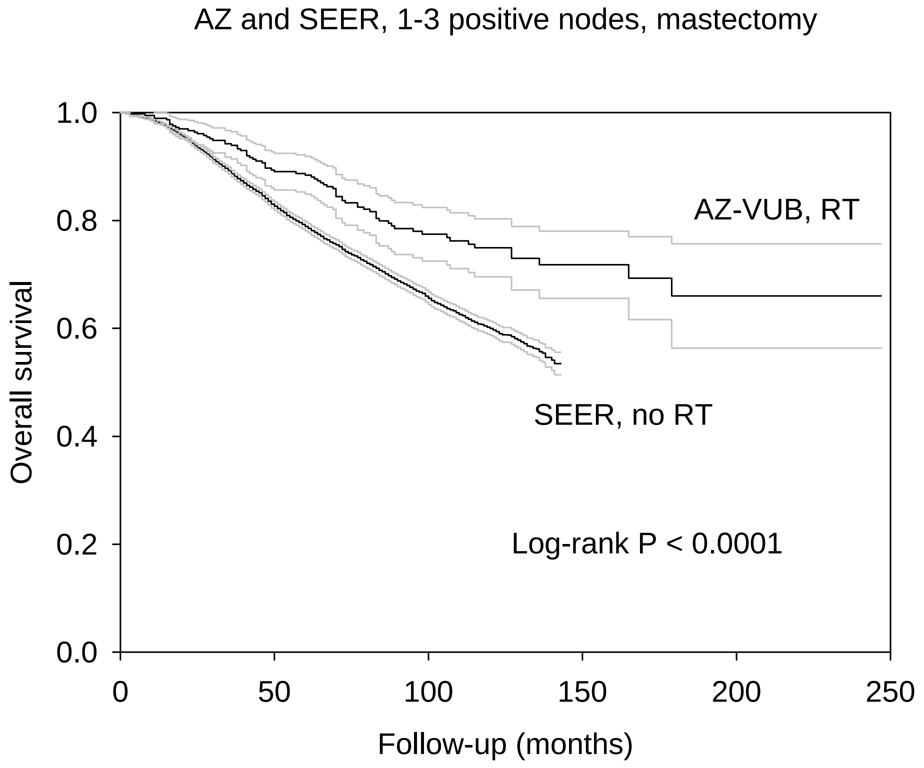

Section 7. Discussion

The results are inconclusive regarding the role of post-mastectomy radiotherapy among node-negative patients, but show a significant survival advantage among node-positive patients, regardless of nodal category. We cannot account for unknown factors such as co-morbidity, social or economic conditions of patients, therefore we cannot exclude potential unknown population differences. However, Section 5 shows the qualitative comparability of SEER and AZ-VUB prognostic factors. Section 6 shows the similarity of survival outcome of breast conserving surgery patients receiving radiation, and the similarity of 4+ node-positive mastectomy patients receiving radiation between SEER and AZ-VUB. These findings argue for the comparability between the patient populations, and argue that differences in survival are attributable to the differences in treatment strategies among some subgroups of patients.

Randomized clinical trials of post-mastectomy were not designed to address the problem of subgroups (*6*). Authors have argued the lack of evidence of a survival advantage in patients with 1-3 positive nodes (*4*), while others found a survival advantage for these patients in randomized clinical trials (*41,42*). Our results are in keeping with the latter findings. It should also be remarked that there is a growing literature that draws the attention to a high rate of local-regional recurrences in the 1-3 positive nodes mastectomy patients who did not receive radiation treatment (*43,44*). While ultimately individual treatment decisions have to take into account other issues like quality of life which was not investigated in this paper, in view of the literature and in view of the present results, the survival outcome and the risk of recurrence need to be discussed whenever post-surgery treatment is considered. In any case, to change our guidelines to omit radiotherapy would not represent progress, but instead a disservice to our patients.

Regarding our secondary objective to identify problems in our own registry, there is an obvious difference in size between the SEER database and our own. With the SEER, one can afford discarding missing records. There are strengths in our registry such as recording details of treatments (not analyzed in the present paper), but the limited size will require improving the quality of follow-up. Limited follow-up restricts the power of survival analyses. Currently tracing of patient status is done from several alternate sources. It might be on the basis on follow-up consultations, but this follow-up can be inexistent or very sparse, e.g. once yearly. Tracing outcome might be based on occasional medical prestation, e.g. a laboratory examination from the family practitioner. On an individual basis, our social department requests last-date of residence or date of death from counties administration where the patient was last known to reside. These are however circumstantial follow-ups that are clearly incomplete, as seen in our short median follow-up of 70 months as compared with the SEER 73 months, despite our longer follow-up range. Linking with billing records might improve our follow-up assessments, but would be applicable only to patients

who are referred to our hospital. Another potential improvement could be a link with the social security reimbursement system in order to trace events, like the SEER-Medicare linkage. This might help improve accuracy of treatment records, though this might bias follow-up towards patients with more healthcare problems. Another alternative would be a direct link with the population counties registries.

Regarding cancer registration in general, there have been some improvement recently with the social security reimbursement for multidisciplinary consults, which provides an incentive for minimal registration of cancer data. It might perhaps evolve in the future towards more detailed registration. But, for the time being, the only source of detailed individual patients cancer data immediately available is the SEER, which explains that it is a reference for any data exploration in cancer. As shown in Table 1, the SEER data was made possible by financial investments. In our case, registration was done on a voluntary basis, which means in fact considerable hidden costs in time rarely available. This clarifies the paradox that we had more facilities with the SEER than with our own patient records.

Appendix

A1. Structure of "Mamma juni 2005" database.
A2. Radiation treatment folder at the AZ-VUB.
A3. WHO histological classification.
A4. Histopathologic grade classification.
A5. TNM classification, reproduced with permission of the College of American Pathologists, http://www.cap.org/apps/docs/cancer_protocols/protocols_index.html, Breast protocol Revision January 2005.

Field Name	Field Type	Formula / Entry Option
AAAAA	Text	Indexed
Adres	Text	Lookup: When an entry in “<unknown>” is made, copy the first matching value from “<Table Missing>”<br><br>If no match: “Do not copy”<br>don't copy “<Table Missing>” if empty
Anapat_i	Text	Indexed, Allow user to override validation, Value List (Custom Values):<br>IDC<br>ILC<br>IDC+is<br>IDC+is+LCIS<br>ILC+is<br>ILC+is+DCIS<br>DCIS<br>LCIS<br>IDC+ILC<br>IDC+ILC+is<br>Sarcoma<br>Medullair<br>IDA - mucineus<br>papillair + IS<br>Andere<br>Onbekend<br>morbus paget
Anapat_i Copy	Text	Indexed, Allow user to override validation, Value List (Custom Values):<br>IDC<br>ILC<br>IDC+is<br>IDC+is+LCIS<br>ILC+is<br>ILC+is+DCIS<br>DCIS<br>LCIS<br>IDC+ILC<br>IDC+ILC+is<br>Sarcoma<br>Medullair<br>IDA - mucineus<br>papillair + IS<br>Andere<br>Onbekend<br>morbus paget
Anapat_i_2	Text	Indexed, Allow user to override validation, Value List (Custom Values):<br>IDC<br>ILC<br>IDC+is<br>IDC+is+LCIS<br>ILC+is<br>ILC+is+DCIS<br>DCIS<br>LCIS<br>IDC+ILC<br>IDC+ILC+is<br>Sarcoma<br>Medullair<br>IDA - mucineus<br>papillair + IS<br>Andere<br>Onbekend<br>morbus paget

Field Name	Field Type	Formula / Entry Option
Anapat_j	Text	
AnapatExtra_i	Text	Indexed
AnapatExtra_j	Text	Indexed
arts code	Text	
BehandelingRecidief_i	Text	Indexed
Bolus	Text	Allow user to override validation, Value List (Custom Values):<br>Y<br>N<br>?
BoostDosis	Text	
Chemo	Text	Indexed, Allow user to override validation, Value List (Custom Values):<br>Postop<br>Preop<br>Preop en postop<br>Radiochemo postop<br>Geen
ChemoType	Text	Indexed, Allow user to override validation, Value List (Custom Values):<br>CMF<br>nvt<br>CEF<br>CAF<br>VNF<br>MMM<br>epirubicine, taxotere<br>Taxol<br>Miton<br>AC<br>epiadria<br>CEF+CMF<br>onbekend<br>CEF x4, Taxol x2<br>TAC<br>Navelbine<br>Herceptine<br>xeloda (trial)
Chirurg	Text	
Complicaties	Text	Indexed
DiffG_BSR_i	Text	Indexed, Allow user to override validation, Value List (Custom Values):<br>?<br>NTB<br>1<br>2<br>3<br>4<br>5
DiffG_BSR_j	Text	Allow user to override validation, Value List (Custom Values):<br>?<br>NTB<br>1<br>2<br>3<br>4<br>5
DiffG_WHO_i	Text	Allow user to override validation, Value List (Custom Values):

Field Name	Field Type	Formula / Entry Option
		?<br>NTB<br>1<br>2<br>3<br>4<br>5
DiffG_WHO_j	Text	Allow user to override validation, Value List (Custom Values):<br>?<br>NTB<br>1<br>2<br>3<br>4<br>5
dossier ok	Text	Indexed, Auto-enter: “”
DossierNr	Text	Indexed
EMD	Text	Indexed
Extern	Text	Lookup: When an entry in “<unknown>” is made, copy the first matching value from “<Table Missing>”<br><br>If no match: “Do not copy”<br>don't copy “<Table Missing>” if empty, Allow user to override validation, Value List (Custom Values):<br>Y<br>N<br>?
Free	Text	Indexed
groene map ok	Text	Indexed, Auto-enter: “”
HKi	Text	Indexed, Allow user to override validation, Value List (Custom Values):<br>Patey<br>Quadrantectomie met axillaire dissectie<br>Mastectomie met sentinel<br>Tumorectomie met sentinel<br>okselevidement post sentinel<br>Patey of Madden<br>Madden<br>Halsted<br>Eenv. Mast.<br>Tumor/quad. ect.<br>Tumorectomie<br>Partiele mastectomie<br>Partiele mastectomie met reperage<br>Partiele mastectomie met echo<br>Bioptie<br>Geen
HKi_def	Text	Indexed, Allow user to override validation, Value List (Custom Values):<br>Patey<br>Quadrantectomie met axillaire dissectie<br>Mastectomie met sentinel<br>Tumorectomie met sentinel<br>okselevidement post sentinel<br>Patey of Madden<br>Madden<br>Halsted<br>Eenv. Mast.

Field Name	Field Type	Formula / Entry Option
		Tumor/quad. ect.<br>Tumorectomie<br>Partiele mastectomie<br>Partiele mastectomie met reperage<br>Partiele mastectomie met echo<br>Bioptie<br>Geen
HKj	Text	
HKj_def	Text	Allow user to override validation, Value List (Custom Values):<br>Patey<br>Quadrantectomie met axillaire dissectie<br>Mastectomie met sentinel<br>Tumorectomie met sentinel<br>okselevidement post sentinel<br>Patey of Madden<br>Madden<br>Halsted<br>Eenv. Mast.<br>Tumor/quad. ect.<br>Tumorectomie<br>Partiele mastectomie<br>Partiele mastectomie met reperage<br>Partiele mastectomie met echo<br>Bioptie<br>Geen
HormonoDosis	Text	
Hormonotherapie	Text	Allow user to override validation, Value List (Custom Values):<br>Postop<br>Preop<br>Preop en postop<br>Radiochemo postop<br>Geen
HormonoType	Text	Allow user to override validation, Value List (Custom Values):<br>nvt<br>TAM<br>Femara+zometa<br>Femara<br>aminogluth.<br>progesteron<br>lentaron<br>MEGACE<br>bil. adnexect.<br>TAM + Zoladex<br>arimidex<br>?<br>onbekend<br>Nolv vrs femara-zometa<br>Tam -> Femara
HUISARTS	Text	Lookup: When an entry in “Mamma juni 2005::arts code” is made, copy the first matching value from “huisartsenDBcode::huisarts”<br><br>If no match: “Copy Next Higher Value”<br><br>don't copy “huisartsenDBcode::huisarts” if empty
Hulp	Text	Allow user to override validation, Value List (Custom Values):<br>Yes

Field Name	Field Type	Formula / Entry Option
		No
ICD	Text	Auto-enter: “174.”
Indicatie_PM_i	Text	Indexed
Indicatie_PM_j	Text	Indexed
JL_Adres HA	Text	
JL_AdresPt	Text	
JL_Anapat	Text	
JL_Chemotherapie	Text	
JL_Chirurgen	Text	
JL_Complicaties	Text	
JL_DCIS	Text	
JL_ERICA	Text	
JL_Follow_Up	Text [10]	
JL_G	Text	
JL_Gemeente patient	Text	
JL_H	Text	
JL_H.	Text	
JL_hormoontherapie	Text	
JL_huidretractie	Text	
JL_ICD pathologie R	Text	
JL_ICDA ingreep R	Text	
JL_Import	Text	
JL_Indicatie t	Text	
JL_Info	Text	
JL_Ingree streaml	Text	
JL_Ingreep	Text	Indexed
JL_Ingreep.XL	Text	Indexed
JL_LocalisatieICd	Text	
JL_LocalisatiePrePost	Text	
JL_Neu_Onco	Text	
JL_pathologie R	Text	
JL_periareolair	Text	
JL_postnummer Pt	Text	
JL_Preop checkup	Text	
JL_Preoperatieve chemo	Text	
JL_PROG	Text	
JL_protocol	Text	
JL_Protocol anapat	Text	
JL_specialist	Text	
JL_tepelretractie	Text	
JL_vasculaire invasie	Text	
Klachten	Text	Indexed
Kompleet	Text	Indexed, Auto-enter: “”, Allow user to override validation, Value List (Custom Values):<br>Y<br>N<br>?

Field Name	Field Type	Formula / Entry Option
Lokaal recidief i	Text	Indexed, Allow user to override validation, Value List (Custom Values):<br><br>Geen<br>Klieren-in velden<br>Klieren-buiten velden<br>Locaal<br>NVT
LRi	Text	Indexed, Allow user to override validation, Value List (Custom Values):<br>L<br>R<br>U<br>B
LRj	Text	Allow user to override validation, Value List (Custom Values):<br>L<br>R<br>U<br>B
menopauzaal	Text	Indexed, Allow user to override validation, Value List (Custom Values):<br>post<br>pre<br>peri<br>?<br>nvt
Metas	Text	Indexed, Allow user to override validation, Value List (Custom Values):<br>Geen<br>Bot<br>Longen<br>Lever<br>Huid<br>Hersenen<br>Pleura<br>Andere
Mi	Text	Indexed
Mj	Text	
Myriam_Aantalveld	Text	Lookup: When an entry in “<unknown>” is made, copy the first matching value from “<Table Missing>”<br><br>If no match: “Do not copy”<br>don't copy “<Table Missing>” if empty
Myriam_Aantalzitt	Text	Lookup: When an entry in “<unknown>” is made, copy the first matching value from “<Table Missing>”<br><br>If no match: “Do not copy”<br>don't copy “<Table Missing>” if empty
Myriam_Meta	Text	Indexed
Myriam_TNM	Text	Lookup: When an entry in “<unknown>” is made, copy the first matching value from “<Table Missing>”<br><br>If no match: “Do not copy”<br>don't copy “<Table Missing>” if empty
Myriam_Toesteltyp	Text	Lookup: When an entry in “<unknown>” is made, copy the first matching value from “<Table Missing>”<br><br>If no match: “Do not copy”<br>don't copy “<Table Missing>” if empty

Field Name	Field Type	Formula / Entry Option
Myriam_Verw_Arts	Text	Lookup: When an entry in “<unknown>” is made, copy the first matching value from “<Table Missing>”<br><br>If no match: “Do not copy”<br>don't copy “<Table Missing>” if empty
Naam	Text	Indexed
NED nazien	Text	Indexed, Auto-enter: “”, Allow user to override validation, Value List (Custom Values):<br>Y<br>N<br>?
Neu	Text	Indexed, Allow user to override validation, Value List (Custom Values):<br><br>neg<br>pos<br>?
Ni	Text	Indexed
NieuwDossierNummer	Text	Lookup: When an entry in “<unknown>” is made, copy the first matching value from “<Table Missing>”<br><br>If no match, use: “????”<br><br>don't copy “<Table Missing>” if empty, Allow user to override validation, Unique values only
Nj	Text	
OestrogeenRec	Text	Indexed, Allow user to override validation, Value List (Custom Values):<br>+++<br>++<br>+<br>-<br>?<br>ntb
Oorspong data	Text	Auto-enter: “Directly entered”, Allow user to override validation, Value List (Custom Values):<br>Directly entered<br>JL<br>RXT
OorzaakOverlijden	Text	Indexed
ParasternaleRX	Text	Allow user to override validation, Value List (Custom Values):<br>Y<br>N<br>?
pMj	Text	
pNi	Text	Indexed, Auto-enter: “”
pNj	Text	
ProgesteronRec	Text	Allow user to override validation, Value List (Custom Values):<br>+++<br>++<br>+<br>-<br>?<br>ntb
protocol AZ	Text	
pTi	Text	Indexed, Auto-enter: “”
pTj	Text	
Quadrant_i	Text	Indexed, Allow user to override validation, Value List (Custom Values):

Field Name	Field Type	Formula / Entry Option
		SE<br>SI<br>IE<br>II<br>C<br>?
Quadrant_j	Text	
RecordOrigin	Text	Indexed, Auto-enter: “Kitty”, Allow user to override validation, Value List (Custom Values):<br>Kitty<br>Lamote<br>Myriam
Regionaal recidief i	Text	Allow user to override validation, Value List (Custom Values):<br>Geen<br>Axillair<br>Susclaviculair<br>Mammaria interna
RTEnergie_i	Text	Indexed
RX_map_Nr	Text	Indexed
SCDosis	Text	
Special	Text	Indexed, Allow user to override validation, Value List (Custom Values):<br>N<br>S<br>D<br>U<br>E<br>M<br>R
Ti	Text	Indexed, Allow user to override validation, Value List (Custom Values):<br>0<br>1<br>2<br>3<br>4<br>x
Tj	Text	
ToDo	Text	Indexed
TweedeTumor	Text	Auto-enter: “”, Allow user to override validation, Value List (Custom Values):<br><br>Vóór borst<br>Na borst<br>nvt
TweedeTumorType	Text	Allow user to override validation, Value List (Custom Values):<br><br>Endometrium<br>colon<br>borst<br>Hodgkin<br>Huid: baso<br>leucemie<br>melanoom<br>long<br>pancreas<br>maag<br>NHL<br>rectum

Field Name	Field Type	Formula / Entry Option
		tongbasis<br>cervix<br>huid-baso<br>ovarium<br>vulva<br>BLAAS<br>NIER<br>KNO<br>galblaas<br>meningeoom
Verw. ziekenhuis	Text	Indexed, Allow user to override validation, Value List (Custom Values):<br>AZVUB<br>Brugmann<br>Bracops<br>Bordet<br>Serruys<br>Ronse<br>Aalst<br>Aalst-Geraardbergen<br>Mechelen<br>Vilvoorde<br>CCDP<br>Clinique du Parc Leopold<br>clinique Edith Cavell<br>Andere<br>UCL<br>st norbertus - Duffel<br>nouvelle clinique de la basilique<br>KUL<br>UZ Gent<br>St. Jan, Brugge<br>Clinique Saint-Remy<br>centre hospitalier etterbeek-Ixelles<br>St. Pierre<br>Erasmus - ULB
VERWIJZEND ARTS	Text	Lookup: When an entry in “Mamma juni 2005::Verw. ziekenhuis” is made, copy the first matching value from “Ziekenhuis_arts LU::DossierNr”<br><br>If no match, use: “”<br><br>don't copy “Ziekenhuis_arts LU::DossierNr” if empty
Search text	Text	Global
AZ-graad	Number	Indexed
ChemoCycli	Number	Auto-enter: “”
Dosis_i	Number	Indexed
JL_#LymfeNN_positief	Number	
JL_%Oestrogeen	Number	
JL_%Progesteron	Number	
JL_%Tumorcellen	Number	
JL_Apex	Number	
JL_Apeximmunohistochemie	Number	
JL_Macrosc_Diam	Number	
JL_Niv I	Number	
JL_Niv II	Number	
JL_Niv III	Number	

Field Name	Field Type	Formula / Entry Option
JL_op apex	Number	
JL_op i	Number	
JL_op ii	Number	
JL_op iii	Number	
JL_op il	Number	
JL_op ing	Number	
JL_op obt	Number	
JL_op posterior superior	Number	
JL_tumor diameter	Number	
N1Pos	Number	
N1Tot	Number	
N2Pos	Number	
N2Tot	Number	
N3Pos	Number	
N3Tot	Number	
N4Pos	Number	
N4Tot	Number	
NTotPos	Number	Indexed
NTotTot	Number	Indexed
OestrogeenRecWaarde	Number	
pMi	Number	
ProgesteronRecWaarde	Number	
StartRTMonth	Number	
StartRTYear	Number	Lookup: When an entry in “<unknown>” is made, copy the first matching value from “<Table Missing>”<br><br>If no match: “Do not copy”<br>don't copy “<Table Missing>” if empty
tumor diameter (cm)	Number	Indexed
Creation date	Date	Indexed, Auto-enter: “Creation Date”
Datum_anapat_i	Date	Indexed, Allow user to override validation, Only allow values of type: “4-Digit Year Date”
Datum_anapat_i_def	Date	Indexed, Auto-enter calculation: = DatumHK_i, Allow user to override validation, Only allow values of type: “4-Digit Year Date”
Datum_anapat_j	Date	Indexed, Allow user to override validation, Only allow values of type: “4-Digit Year Date”
datum_HK_def	Date	Allow user to override validation, Only allow values of type: “4-Digit Year Date”
datum_HKj	Date	Allow user to override validation, Only allow values of type: “4-Digit Year Date”
Datum_laatste_bericht_in_lev en	Date	Indexed
DatumHK_i	Date	Indexed, Auto-enter calculation: = Datum_anapat_i, Allow user to override validation, Only allow values of type: “4-Digit Year Date”
DatumMetas	Date	Indexed, Allow user to override validation, Only allow values of type: “4-Digit Year Date”
DatumRecidief_i	Date	Indexed, Auto-enter: “”, Allow user to override validation, Only allow values of type: “4-Digit Year Date”
EMD2	Date	Indexed, Allow user to override validation, Only allow values of type: “4-Digit Year Date”

Field Name	Field Type	Formula / Entry Option
InvoegDatum	Date	Auto-enter: “Modification Date”
JL_Occurrence date	Date [10]	
LaatsteCons	Date	Lookup: When an entry in “<unknown>” is made, copy the first matching value from “<Table Missing>”<br><br>If no match: “Do not copy”<br>don't copy “<Table Missing>” if empty
Last modification	Date	Auto-enter: “Modification Date”
Overleden	Date	Indexed, Allow user to override validation, Only allow values of type: “4-Digit Year Date”
StartRT_i	Date	Indexed, Allow user to override validation, Only allow values of type: “4-Digit Year Date”
StopHormono	Date	Auto-enter: “”, Allow user to override validation, Only allow values of type: “4-Digit Year Date”
StopRT_i	Date	Indexed, Allow user to override validation, Only allow values of type: “4-Digit Year Date”
Achternaam	Calculation (Text)	Indexed, = Choose(WordCount(Naam);""; Naam; LeftWords(Naam; 1); LeftWords(Naam; 2))
Dossier7Naam	Calculation (Text)	Indexed, = DossierNr11 & Naam
DossierNr11	Calculation (Text)	Indexed, = Middle(DossierNr; 1; 11)
Ook naam	Calculation (Text)	Indexed, = Naam, Evaluate even if all referenced fields are empty
UniqueText	Calculation (Text)	Indexed, = DossierNr11&Naam
ValidDossierNr	Calculation (Text)	= If(IsValid(DossierNr); "";"not a date, ") & If(Length(DossierNr)=12; ""; "length is not 12 characters, ") & If(((Left(DossierNr;1) ="a") or (Left(DossierNr;1) ="b")); ""; "doesn't start with 'a' or 'b', ")
Comparison result	Calculation (Number)	Unstored, = Exact(JL_Ingreep;Search text)
Delay	Calculation (Number)	= Duur_i - (7*Dosis_i/2*5)
Died	Calculation (Number)	Indexed, = If(IsEmpty(Overleden); 0; 1), Evaluate even if all referenced fields are empty
DossierNrOK	Calculation (Number)	Indexed, = Middle(DossierNr; 1; 1) = "B" and Middle(DossierNr; 2; 1) ≥ "0"and Middle(DossierNr; 2; 1) ≤ "9"and Middle(DossierNr; 3; 1) ≥ "0"and Middle(DossierNr; 3; 1) ≤ "9"and Middle(DossierNr; 4; 1) ≥ "0"and Middle(DossierNr; 4; 1) ≤ "9"and Middle(DossierNr; 5; 1) ≥ "0"* Middle(DossierNr; 5; 1) ≤ "9"and Middle(DossierNr; 6; 1) ≥ "0"and Middle(DossierNr; 6; 1) ≤ "9"and Middle(DossierNr; 7; 1) ≥ "0"and Middle(DossierNr; 7; 1) ≤ "9" + Length(DossierNr)>=11
Duur_i	Calculation (Number)	= StopRT_i-StartRT_i
FU_Moths	Calculation (Number)	= If((GetAsNumber(Died)=0); (Laatste_Datum-Datum_anapat_i_def)/30.5; (Overleden-Datum_anapat_i_def)/30.5)
LeeftijdBijDiagnose	Calculation (Number)	Indexed, = If(Round(((Datum_anapat_i-GeboortedatumCalc-182.5) /365.25);0)<10; Round(((Datum_anapat_i-GeboortedatumCalc-182.5) /365.25);0) +100; Round(((Datum_anapat_i-GeboortedatumCalc-182.5) /365.25);0))
LenDossierNr	Calculation (Number)	Indexed, = Length(DossierNr)
Temp	Calculation (Number)	Unstored, = (not ((IsEmpty(<Table Missing>::<Field Missing>)) and (IsEmpty(Kompleet))))
GeboortedatumCalc	Calculation (Date)	= GetAsDate(Middle(DossierNr; 6; 2) & "-" & Middle(DossierNr; 4; 2) & "-" & Middle(DossierNr; 2; 2))
Laatste_Datum	Calculation (Date)	= Max(DatumHK_i;DatumMetas;DatumRecidief_i;Datum_anapat_i; Datum_anapat_i_def;Datum_anapat_j;datum_HKj;datum_HK_def; LaatsteCons;Datum_laatste_bericht_in_leven;StartRT_i;StopRT_i; StopHormono)

Field Name	Field Type	Formula / Entry Option
MaxLftdBijDiagnose	Summary (Number)	= Maximum of LeeftijdBijDiagnose
MinLftdBijDiagnose	Summary (Number)	= Minimum of LeeftijdBijDiagnose

ONCOLOGISCH CENTRUM **AZ-VUB** **DEPT.RADIOTHERAPIE**

Nr. simulatiefiche: .... **SIMULATIEFICHE** Linac: .... Kaftnr: ....

Patientenidentificatie

Telefoon: ..../............................

PS: (Karnofski)

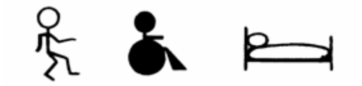

[ ]

Dossiernr. radiotherapie: ..../....................

Accepterende arts: ....

Verwijzing: Intern O Extern O

Verwijzend arts: ....

Ziekenhuis: ....

Huisarts: ....

Datum simulatie: ..../........../..........

Simulatiearts: ....

Startdatum therapie: ..../........../..........

Voorziene einddatum: ..../........../..........

Effectieve einddatum: ..../........../..........

DIAGNOSE:

Primair letsel: ....

Doelvolume: ....

....

ICD-code: ....

PA.uitslag: ....

....

....

cT [ ] **N** [ ] **M** [ ] **STADIUM:**........

pT [ ] **N** [ ] **M** [ ] **STADIUM:**.......

Meta: ....

Voorgaande behandeling: ....

....

....

....

....

Volgende reeks(en): JA O NEEN O

⇒ Externe radioth. O BRACHY. O

Fractionatie 2de reeks: .... X .... Gy

Fractionatie 3de reeks: .... X .... Gy

Rustperiode: ....

Opmerkingen: ....

....

BEHANDELINGSSCHEMA

CODE PROTOCOL: .... STUDIEPROTOCOL: ....

Totaal dosis: ....Gy	Fractionatie: ....X..........Gy	Fracties/dag: ....
Voorgaande dosis: ....Gy	Aantal veld./fractie:	Fracties/week: ....

**DOSIS GY**	APPA/ PA+LAT	LAT	S.C.	TANG/ SCHUIN	CROSS/ BOX	CFR. 3D-PLANNING			BRACHY

X:start **S**:stoppen **F**:wijzigen fractiedosis **E**:evaluatie **V**:veldverkl.-wijziging **B**:blokk.verand. **J**:jawsaanpassing

PARAF: RESIDENT ASSISTENT VERPLEGENDE KONTR. VELDINST. ARTS

① ② ③

TUMORLOKALISATIE EN VELDAANDUIDING

**Toestel:**	Linac 1	O	Fotonen:	O	........MV	**Techniek:**	SSD	O	STAT	O
	Linac 3	O	Electronen:	O	........MeV		SAD	O	STX	O
						ANDERE:				

Positionering:

Ruglig	O	Arntli	O
Buiklig	O	Arbvhfd	O
Zijlig	O	Opschtr	O
Hfdnrre	O	Driem	O
Hfdnrli	O	Volle blaas	O
Vtnrgtr	O	Lege blaas	O
Aroth	O	Bolus	O

Hulpstukken:

Neknr:	....	Oordopjes	O
Nekopbouw	.... cm	Longsteun: X Y Z	O
Nekspie:	**1** O **2** O	Borststeun: X Y Z	O
Aantal kss.hfd:	O	Helling : K L M	O
Rugspie	O	Vacuummatras	O
Prone-pillow	O	Gelkss.	O
Masker	O	Klkshi Le	O
Tongdepressor	O	Klkshi Br	O
Liptampons	O	Knierol	O
Armsteun Li	A B C D E	Armsteun A B C D E	
Armsteun Re	A B C D E		

Andere: ....

....

Plaatsing Nekspie **1** = brede zijde -> craniaal **2** = brede zijde-> caudaal

**Bolus:** dikte: ....mm<br>materiaal: ....<br>..<br>lokalisatie:	**Opmerkingen:**<br>...<br>...<br>...

PLANNING

NEEN O

JA O ⇒

GT O 3D O

3D-CRT O ⇒ Radiotherapeut: ....
⇒ Fysicus: ....

Datum CT: ..../......./....... Planning uit te voeren op bestaande CT O

SIM-CT O Voorstel veldwijziging: O ....Gy

Planvoorstel na aslokal.: O ⇒ voorziene simul.datum: ..../......./........

Kwaliteitskontrole:

		Dosis	Item	Datum / Paraf
Portal	O			
	O			
	O			
	O			
Epi	O			
Epi	O			
TLD	O			
Diode	O			

**Onderzoeken:**	Datum Onderzoek	Paraf + Datum

Datum:	**Konsultatie Dietiste**

Gewichtstabel:

Datum	Gewicht	Datum	Gewicht	Datum	Gewicht
	Kg		Kg		Kg
	Kg		Kg		Kg
	Kg		Kg		Kg
	Kg		Kg		Kg

**Konsultatie te voorzien bij:**		Frekwentie:			Paraf:
Sociaal Verplegende:	O	Begin behandeling	O	....	
Dietiste:	O	Begin behandeling	O	....	
Radiotherapeut:	O	....		....	
Arts: KNO.	O	....		Data: ...../....., .../....., .../.....,	
				...../....., .../....., .../....., .../.....	
Arts:	O	....		....	
Arts:	O	....		....	
Psychologe:	O	....		....	
....	O	....		....	

DATUM	VERPLEEGKUNDIGE AANDACHTSPUNTEN	PARAF VERPL.

A3. WHO histological classification of tumours of the breast (*45*)

**Epithelial tumours**	
Invasive ductal carcinoma, not otherwise specified (*)	8500/3
Mixed type carcinoma	
Pleomorphic carcinoma	8022/3
Carcinoma with osteoclastic giant cells	8035/3
Carcinoma with choriocarcinomatous features	
Carcinoma with melanotic features	
Invasive lobular carcinoma (*)	8520/3
Tubular carcinoma	8211/3
Invasive cribriform carcinoma	8201/3
Medullary carcinoma	8510/3
Mucinous carcinoma and other tumours with abundant mucin	
Mucinous carcinoma	8480/3
Cystadenocarcinoma and columnar cell mucinous carcinoma	8480/3
Signet ring cell carcinoma	8490/3
Neuroendocrine tumours	
Solid neuroendocrine carcinoma	
Atypical carcinoid tumour	8249/3
Small cell / oat cell carcinoma	8041/3
Large cell neuroendocrine carcinoma	8013/3
Invasive papillary carcinoma	8503/3
Invasive micropapillary carcinoma	8507/3
Apocrine carcinoma	8401/3
Metaplastic carcinomas	8575/3
Pure epithelial metaplastic carcinomas	8575/3
Squamous cell carcinoma	8070/3
Adenocarcinoma with spindle cell metaplasia	8572/3
Adenosquamous carcinoma	8560/3
Mucoepidermoid carcinoma	8430/3
Mixed epithelial/mesenchymal metaplastic carcinomas	8575/3
Lipid-rich carcinoma	8314/3
Secretory carcinoma	8502/3
Oncocytic carcinoma	8290/3
Adenoid cystic carcinoma	8200/3
Acinic cell carcinoma	8550/3
Glycogen-rich clear cell carcinoma	8315/3
Sebaceous carcinoma	8410/3
Inflammatory carcinoma	8530/3
Lobular neoplasia	
Lobular carcinoma in situ	8520/2
Intraductal proliferative lesions	
usual ductal hyperplasia	
Flat epithelial atypia	
Atypical ductal hyperplasia	
Ductal carcinoma in situ	8500/2
Microinvasive carcinoma	
Intraductal papillary neoplasms	
Central papilloma	8503/0
Peripheral papilloma	8503/0
Atypical papilloma	
Intraductal papillary carcinoma	8503/2
Intracystic papillary carcinoma	8504/2
Benign epithelial proliferations	
Adenosis including variants	
Sclerosing adenosis	
Apocrine adenosis	
Blunt duct adenosis	
Microglandular adenosis	
Adenomyoepithelial adenosis	
Radial scar/ complex sclerosing lesion	

Adenomas	
Tubular adenoma	8211/0
Lactating adenoma	8204/0
Apocrine adenoma	8401/0
Pleomorphic adenoma	8940/0
Ductal adenoma	8503/0
**Myoepithelial lesions**	
Myoepitheliosis	
Adenomyoepithelial adenosis	
Adenomyoepithelioma	8983/0
Malignant myoepithelioma	8982/3
**Mesenchymal tumours**	
Haemangioma	9120/0
Angiomatosis	
Haemangiopericytoma	9150/1
Pseudoangiomatous stromal hyperplasia	
Myofibroblastoma	8825/0
Fibromatosis (aggressive)	8821/1
Inflammatory myofibroblastic tumour	8825/1
Lipoma	8850/0
Angiolipoma	8861/0
Granular cell tumour	9580/0
Neurofibroma	9540/0
Schwannoma	9560/0
Angiosarcoma	9120/3
Liposarcoma	8850/3
Rhabdomyosarcoma	8900/3
Osteosarcoma	9180/3
Leiomyoma	8890/0
Leiomyosarcoma	8890/3
**Fibroepithelial tumours**	
Fibroadenoma	9010/0
Phyllodes tumour	9020/1
Benign	9020/0
Borderline	9020/1
Malignant	9020/3
Periductal stromal sarcoma, low grade	9020/3
Mammary hamartoma	
**Tumours of the nipple**	
Nipple adenoma	8506/0
Syringomatous adenoma	8407/0
Paget disease of the nipple	8540/3
**Malignant lymphoma**	
Diffuse large B-cell lymphoma	9680/3
Burkitt lymphoma	9687/3
Extranodal marginal-zone B-cell lymphoma of MALT type	9699/3
Follicular lymphoma	9690/3
**Metastatic tumours**	
**Tumours of the male breast**	
Gynaecomastia	
Carcinoma	
Invasive	8500/3
In situ	8500/2

Malignant tumours (cancers) are coded /3. In situ carcinomas and grade 3 intraepithelial neoplasia are coded /2. Benign tumours are coded /0. Borderline tumours or uncertain behaviour are coded /1.

(*) Mixed invasive ductal and lobular carcinoma coded as 8522/3.

A4. Histologic Grade based on the Nottingham Histologic Score (*46*)

Feature	Score
Tubule and gland formation	
___ Majority of tumor greater than 75%	1
___ Moderate 10% to 75%	2
___ Minimal less than 10%	3
Nuclear Pleomorphism	
___ Small regular nuclei	1
___ Moderate increase in size and variability	2
___ Marked variation	3
Mitotic Count	
For a 25x objective with a field area of 0.274 mm^2	
___ Less than 10 mitoses per 10 HPF	1
___ 10 to 20 mitoses per 10 HPF	2
___ Greater than 20 mitoses per 10 HPF	3
or	
For a 40x objective with a field area of 0.152 mm^2	
___ 0 to 5 mitoses per 10 HPF	1
___ 6 to 10 mitoses per 10 HPF	2
___ Greater than 10 mitoses per 10 HPF	3
Total Nottingham Score	
___ Grade 1 – well differentiated: 3-5 points	
___ Grade 2 – moderately differentiated: 6-7 points	
___ Grade 3 – poorly differentiated: 8-9 points	

A5. TNM classification of carcinomas of the breast (*47,48*)

Primary Tumor (cT and pT)

TX	Primary tumor cannot be assessed[a]
T0	No evidence of primary tumor
Tis	Carcinoma in situ: ductal carcinoma in situ (DCIS), lobular carcinoma in situ (LCIS), or Paget disease of the nipple with no tumor[b]
T1	Tumor 2 cm or less in greatest dimension
T1mic	Microinvasion 0.1 cm or less in greatest dimension[c]
T1a	Tumor more than 0.1 cm but not more than 0.5 cm in greatest dimension
T1b	Tumor more than 0.5 cm but not more than 1 cm in greatest dimension
T1c	Tumor more than 1 cm but not more than 2 cm in greatest dimension
T2	Tumor more than 2 cm but not more than 5 cm in greatest dimension
T3	Tumor more than 5 cm in greatest dimension
T4	Tumor of any size with direct extension to chest wall or skin,[d] but only as described below
T4a	Extension to chest wall, not including pectoralis muscle[e]
T4b	Edema (including peau d'orange) or ulceration of the skin of the breast or satellite skin nodules confined to the same breast
T4c	Both T4a and T4b
T4d	Inflammatory carcinoma[f]

[a] If tumor is present at the margin of the resection by macroscopic examination, case coded as pTX because the total extent of tumor cannot be assessed.
[b] Paget disease associated with a tumor is classified according to the size of the tumor.
[c] Microinvasion is extension of cancer cells beyond the basement membrane into adjacent tissues with no focus more than 0.1 cm in greatest dimension. When there are multiple foci of microinvasion, the size of only the largest focus is used to classify the microinvasion (do not use the sum of all the individual foci). The presence of multiple foci should be noted and/or quantified, as with multiple larger invasive carcinomas.
[d] Dermal invasion alone (without ulceration, satellite nodules, or inflammatory breast cancer) does not alter T category. Such cases are classified as T1, T2, or T3, depending on tumor size.
[e] Tumor in pectoral muscle should be measured with the breast tumor for determining tumor size and final T category.
[f] Inflammatory carcinoma of the breast is a clinicopathologic entity characterized by diffuse erythema and edema involving the majority of the skin of the breast, often without an underlying palpable mass. The clinical presentation is due to tumor emboli in dermal lymphatics, although these may not be seen on skin biopsy. The diagnosis is established by the combination of the clinical findings and a biopsy showing cancer, either within dermal lymphatics or in the breast parenchyma. Involvement of dermal lymphatics alone does not indicate inflammatory carcinoma. If the skin biopsy is negative and there is no localized measurable primary cancer, the T category is pTX when pathologically staging a clinical inflammatory carcinoma (T4d). Dimpling of the skin, nipple retraction, or other skin changes, except those in T4b and T4d, may occur in T1, T2, or T3 without affecting the classification.

Regional Lymph Nodes (cN)#

NX	Cannot be assessed (e.g. previously removed)
N0	No regional lymph node metastasis
N1	Metastasis in movable ipsilateral axillary lymph node(s)
N2	Metastasis in fixed ipsilateral axillary lymph node(s) *or* in clinically apparent* ipsilateral internal mammary nodes in the *absence* of clinically evident axillary lymph node metastasis
N2a	Metastasis in axillary lymph node(s) fixed to one another or to other structures
N2b	Metastasis only in clinically apparent* internal mammary lymph node(s) in the *absence* of clinically evident axillary lymph node metastasis
N3	Metastasis in ipsilateral infraclavicular lymph node(s) with or without axillary lymph node involvement; or in clinically apparent* ipsilateral internal mammary lymph node(s) in the presence of clinically evident axillary lymph node metastasis; or metastasis in ipsilateral supraclavicular lymph node(s) with or without axillary or internal mammary lymph node involvement
N3a	Metastasis in infraclavicular lymph node(s)
N3b	Metastasis in internal mammary and axillary lymph nodes
N3c	Metastasis in supraclavicular lymph node(s)

The regional lymph nodes are:
1. Axillary (ipsilateral): interpectoral (Rotter) nodes and lymph nodes along the axillary vein and its tributaries, which may be divided into following levels:
(i) Level I (low-axilla): lymph nodes lateral to the lateral border of pectoralis minor muscle.
(ii) Level II (mid-axilla): lymph nodes between medial and lateral borders of the pectoralis minor muscle, and interpectoral (Rotter) lymph nodes.
(iii) Level III (apical axilla): apical lymph nodes and those medial to the medial margin of the pectoralis minor muscle, excluding those designated as subclavicular or infraclavicular.
Note: Intramammary lymph nodes are coded as axillary lymph nodes, level I.
2. Infraclavicular (subclavicular) (ipsilateral).
3. Internal mammary (ipsilateral): lymph nodes in the intercostal spaces along the edge of the sternum in the endothoracic fascia.
4. Supraclavicular (ipsilateral).
* clinically apparent = detected by clinical examination or by imaging studies, xcluding lymphoscintigraphy)

Regional Lymph Nodes (pN)[a]

pNX	Cannot be assessed (previously removed or not removed for pathologic study)
pN0	No regional lymph node metastasis histologically, no examination for isolated tumor cells (ITCs)[b]
pN0(i-)	No regional lymph node metastasis histologically, negative morphologic (any morphologic technique, including hematoxylin-eosin and immunohistochemistry) findings for ITCs
pN0(i+)	No regional lymph node metastasis histologically, positive morphologic (any morphologic technique, including hematoxylin-eosin and immunohistochemistry) findings for ITCs, no ITC cluster greater than 0.2 mm
pN0(mol-)	No regional lymph node metastasis histologically, negative nonmorphologic (molecular) findings for ITCs
pN0(mol+)	No regional lymph node metastasis histologically, positive nonmorphologic (molecular) findings for ITCs
pN1	Metastasis in 1 to 3 axillary lymph nodes, and/or internal mammary nodes with microscopic disease detected by sentinel lymph node dissection but not clinically apparent[c]
pN1mi	Micrometastasis (greater than 0.2 mm, none greater than 2.0 mm)
pN1a	Metastasis in 1 to 3 axillary lymph nodes (at least 1 tumor deposit greater than 2.0 mm)
pN1b	Metastasis in internal mammary nodes with microscopic disease detected by sentinel lymph node dissection but not clinically apparent[c]
pN1c	Metastasis in 1 to 3 axillary lymph nodes and in internal mammary nodes with microscopic disease detected by sentinel lymph node dissection but not clinically apparent. (If associated with more than 3 positive axillary lymph nodes, the internal mammary nodes are classified as pN3b to reflect increased tumor burden)
pN2	Metastasis in 4 to 9 axillary lymph nodes or in clinically apparent[c] internal mammary nodes in the absence of axillary lymph node metastasis

pN2a Metastasis in 4 to 9 axillary lymph nodes (at least 1 tumor deposit larger than 2.0 mm)
pN2b Metastasis in clinically apparent[c] internal mammary nodes in the absence of axillary lymph node metastasis
pN3 Metastasis in 10 or more axillary lymph nodes, or in infraclavicular lymph nodes, or in clinically apparent[c] ipsilateral internal mammary nodes in the presence of 1 or more positive axillary lymph nodes; or in more than 3 axillary lymph nodes with clinically negative microscopic metastasis in internal mammary nodes or in ipsilateral supraclavicular lymph nodes
pN3a Metastasis in 10 or more axillary lymph nodes (at least 1 tumor deposit greater than 2.0 mm), or metastasis to the infraclavicular lymph nodes
pN3b Metastasis in clinically apparent[c] ipsilateral internal mammary lymph nodes in the presence of 1 or more positive axillary lymph nodes; or in more than 3 axillary lymph nodes and in internal mammary nodes with microscopic disease detected by sentinel lymph node dissection but not clinically apparent.
pN3c Metastasis in ipsilateral supraclavicular lymph nodes

There are instances when the pathologist cannot make this determination because the complete staging procedure, such as a lymph node dissection, has not been performed or because information about a prior procedure is unavailable. In such situations "X" is used rather than a number in the TNM designation.
[a] Classification is based on axillary lymph node dissection with or without sentinel lymph node dissection. Classification based solely on sentinel lymph node dissection without subsequent axillary dissection is designated (sn) for "sentinel node," eg, pN0(i+)(sn).
[b] Isolated tumor cells (ITC) are defined as single tumor cells or small cell clusters not greater than 0.2 mm. They may be detected by routine histologic examination or by immunohistochemical (IHC) or molecular methods. ITCs do not usually show evidence of malignant activity (eg, proliferation or stromal reaction).
[c] Clinically apparent is defined as detected by imaging studies (excluding lymphoscintigraphy) or by clinical examination. Not clinically apparent is defined as not detected by imaging studies (excluding lymphoscintigraphy) or by clinical examination.
[d] Micrometastases may show histologic evidence of malignant activity (eg, proliferation or stromal reaction).

Distant Metastasis (cM and pM)
MX Presence of distant metastasis cannot be assessed
M0 No distant metastasis
M1 Distant metastasis

TNM Descriptors
For identification of special cases of TNM or pTNM classifications, the "m" suffix and "y," "r," and "a" prefixes are used. Although they do not affect the stage grouping, they indicate cases needing separate analysis.
The "m" suffix indicates the presence of multiple primary tumors in a single site and is recorded in parentheses: pT(m)NM.
The "y" prefix indicates those cases in which classification is performed during or following initial multimodality therapy (ie, neoadjuvant chemotherapy, radiation therapy, or both chemotherapy and radiation therapy). The cTNM or pTNM category is identified by a "y" prefix. The ycTNM or ypTNM categorizes the extent of tumor actually present at the time of that examination. The "y" categorization is not an estimate of tumor prior to multimodality therapy (ie, before initiation of neoadjuvant therapy).
The "r" prefix indicates a recurrent tumor when staged after a documented disease-free interval, and is identified by the "r" prefix: rTNM.
The "a" prefix designates the stage determined at autopsy: aTNM.

Additional Descriptors

Residual Tumor (R)
Tumor remaining in a patient after therapy with curative intent (eg, surgical resection for cure) is categorized by a system known as R classification.
RX Presence of residual tumor cannot be assessed
R0 No residual tumor
R1 Microscopic residual tumor
R2 Macroscopic residual tumor
For the surgeon, the R classification may be useful to indicate the known or assumed status of the completeness of a surgical excision. For the pathologist, the R classification is relevant to the status of the margins of a surgical resection specimen. That is, tumor involving the resection margin on pathologic examination may be assumed to correspond to residual tumor in the patient and may be classified as macroscopic or microscopic according to the findings at the specimen margin(s).

Vessel Invasion
By AJCC/UICC convention, vessel invasion (lymphatic or venous) does not affect the T category indicating local extent of tumor unless specifically included in the definition of a T category. In all other cases, lymphatic and venous invasion by tumor are coded separately as follows:

Lymphatic (Small Vessel) Invasion (L)
LX Lymphatic vessel invasion cannot be assessed
L0 No lymphatic vessel invasion
L1 Lymphatic vessel invasion

Venous (Large Vessel) Invasion (V)
VX Venous invasion cannot be assessed
V0 No venous invasion
V1 Microscopic venous invasion
V2 Macroscopic venous invasion

Regional Lymph Nodes (pN0): Isolated Tumor Cells
Isolated tumor cells (ITC) are single cells or small clusters of cells not more than 0.2 mm in greatest dimension. Lymph nodes or distant sites with ITC found by either histologic examination, immunohistochemistry, or nonmorphologic techniques (eg, flow cytometry, DNA analysis, polymerase chain reaction [PCR] amplification of a specific tumor marker) should be classified as N0 or M0, respectively. Specific denotation of the assigned N category is suggested as follows for cases in which ITC are the only evidence of possible metastatic disease.
pN0 No regional lymph node metastasis histologically, no examination for isolated tumor cells (ITCs)
pN0(i-) No regional lymph node metastasis histologically, negative morphologic (any morphologic technique, including hematoxylin-eosin and immunohistochemistry) findings for ITCs
pN0(i+) No regional lymph node metastasis histologically, positive morphologic (any morph. techn., including h-e and immunohis.) findings for ITCs
pN0(mol-) No regional lymph node metastasis histologically, negative nonmorphologic (molecular) findings for ITCs
pN0(mol+) No regional lymph node metastasis histologically, positive nonmorphologic (molecular) findings for ITCs

Sentinel Lymph Nodes
The sentinel lymph node is the first node to receive drainage from a primary tumor. There may be more than 1 sentinel node for some tumors. If a sentinel node contains metastatic tumor, it indicates that other more distant nodes may also contain metastatic disease. If sentinel nodes are negative, other regional nodes are less likely to contain metastasis. Sentinel lymph nodes that have been examined for ITCs are denoted as follows:
pN0(sn) No sentinel lymph node metastasis histologically (ie, none greater than 0.2 mm), no additional examination for isolated tumor cells (ITCs)
pN0(i-)(sn) No sentinel lymph node metastasis histologically (ie, none greater than 0.2 mm), negative morphologic findings for ITCs
pN0(i+)(sn) No sentinel lymph node metastasis histologically, positive morphologic findings for ITCs
pN0(mol-)(sn) No sentinel lymph node metastasis histologically, negative nonmorphologic findings for ITCs
pN0(mol+)(sn) No sentinel lymph node metastasis histologically, positive nonmorphologic findings for ITCs

References

(1) Parkin DM, Whelan SL, Ferlay J, Storm H. Cancer Incidence in Five Continents, Vol. I to VIII. IARC CancerBase No 7 2005;No. 7: Available at http://www-dep.iarc.fr/. [Last accessed: 6-28-2005.]

(2) Belgisch Werk Tegen Kanker. Fiches 1998: Borstkanker. Belgisch Werk Tegen Kanker 2003; Available at http://www.webweaver.be/nkr/www/fiches1998/borstkanker.pdf. [Last accessed: 8-29-2005.]

(3) Scarth H, Cantin J, Levine M. Clinical practice guidelines for the care and treatment of breast cancer: mastectomy or lumpectomy? The choice of operation for clinical stages I and II breast cancer (summary of the 2002 update). CMAJ 2002;167:154-5.

(4) Recht A, Edge SB, Solin LJ, Robinson DS, Estabrook A, Fine RE, et al. Postmastectomy radiotherapy: clinical practice guidelines of the American Society of Clinical Oncology. J Clin Oncol 2001;19:1539-69.

(5) Vinh-Hung V, Verschraegen C, The Breast Conserving Surgery Project. Breast-conserving surgery with or without radiotherapy: pooled-analysis for risks of ipsilateral breast tumor recurrence and mortality. J Natl Cancer Inst 2004;96:115-21. Available at http://jncicancerspectrum.oupjournals.org/cgi/content/full/jnci;96/2/115. [Last accessed: 3-27-2005.]

(6) Early Breast Cancer Trialists' Collaborative Group (EBCTCG). Favourable and unfavourable effects on long-term survival of radiotherapy for early breast cancer: an overview of the randomised trials. Lancet 2000;355:1757-70.

(7) Gaffney DK, Tsodikov A, Wiggins CL. Diminished survival in patients with inner versus outer quadrant breast cancers. J Clin Oncol 2003;21:467-72.

(8) Vinh-Hung V, Burzykowski T, Van de Steene J, Voordeckers M, Lamote J, Storme G. Statistical interaction in the survival analysis of early breast cancer using registry data: role of breast conserving surgery and radiotherapy. Tumori 2005;91:9-14. Available at http://www.tumorionline.it/numeri/pdf/1_05/03-Vinh-Hung%20(9-14).pdf. [Last accessed: 3-27-2005.]

(9) Van de Steene J, Soete G, Storme G. Adjuvant radiotherapy for breast cancer significantly improves overall survival: the missing link. Radiother Oncol 2000;55:263-72.

(10) Van de Steene J, Vinh-Hung V, Storme G. Longer follow-up of randomized trials on adjuvant radiotherapy for breast cancer stresses the overall survival benefit due to radiotherapy. Abstract 196. Int J Radiat Oncol Biol Phys 2001;51 Suppl 1:109-10.

(11) Van de Steene J, Vinh-Hung V, Cutuli B, Storme G. Adjuvant radiotherapy for breast cancer: effects of longer follow-up. Radiother Oncol 2004;72:35-43.

(12) Storme G, Teppo L, Coebergh JW, Van den Berge D, Vinh-Hung V. Improved survival in breast cancer during the 80's can largely be explained by earlier diagnosis. Abstract 539. Proc Am Soc Clin Oncol 2000;19:137a.

(13) Vinh-Hung V, Burzykowski T, Van de Steene J, Storme G, Soete G. Post-surgery radiation in early breast cancer: survival analysis of registry data. Radiother Oncol 2002;64:281-90.

(14) Vinh-Hung V, Voordeckers M, Van de Steene J, Soete G, Lamote J, Storme G. Omission of radiotherapy after breast-conserving surgery: survival impact and time trends. Radiother Oncol 2003;67:147-58.

(15) Vinh-Hung V, Cserni G, Burzykowski T, Van de Steene J, Voordeckers M, Storme G. Effect of the number of uninvolved nodes on survival in early breast cancer. Oncol Rep 2003;10:363-8. Available at

http://147.52.72.117/OR/2003/volume10/number2/363.pdf. [Last accessed: 7-11-2004.]

(16) Vinh-Hung V, Burzykowski T, Cserni G, Voordeckers M, Van de Steene J, Storme G. Functional form of the effect of the numbers of axillary nodes on survival in early breast cancer. Int J Oncol 2003;22:697-704. Available at http://147.52.72.117/IJO/2003/volume22/number3/697.pdf. [Last accessed: 7-11-2004.]

(17) Vinh-Hung V, Van de Steene J, Cserni G, Vlastos G, Voordeckers M, Storme G. Modeling the effect of age in T1-T2 breast cancer. Abstract 405. Eur J Cancer Suppl 2003;1:S123.

(18) Collen C, Fontaine C, Samijn I, Vlastos G, Tai P, Bretz A, et al. Long-term prognostic value of oestrogen receptor and progesterone receptor in early stage breast cancer. Abstract S5. Eur J Cancer Suppl 2004;2:89.

(19) Tai P, Yu E, Vinh-Hung V, Cserni G, Vlastos G. Survival of patients with metastatic breast cancer: twenty-year data from two SEER registries. BMC Cancer 2004;4:60.

(20) Vinh-Hung V, Storme G. Gompertzian effect of tumor size on mortality in early breast cancer. Abstract 391. Eur J Cancer Suppl 2004;2:170.

(21) Verschraegen C, Vinh-Hung V, Cserni G, Gordon R, Royce ME, Vlastos G, et al. Modeling the effect of tumor size in early breast cancer. Ann Surg 2005;241:309-18.

(22) Tai P, Cserni G, Van de Steene J, Vlastos G, Voordeckers M, Royce M, et al. Modeling the Effect of Age in T1-2 Breast Cancer using the SEER Database. BMC Cancer 2005;(in press):

(23) Voordeckers M, Van de Steene J, Vinh-Hung V, Storme G. Adjuvant radiotherapy after mastectomy for pT1-pT2 node negative (pN0) breast cancer: is it worth the effort? Radiother Oncol 2003;68:227-31.

(24) Voordeckers M, Vinh-Hung V, Van de Steene J, Lamote J, Storme G. The lymph node ratio as prognostic factor in node-positive breast cancer. Radiother Oncol 2004;70:225-30.

(25) Hewitt M, Simone JV. Enhancing data systems to improve the quality of cancer care. Washington, D.C.: National Academy Press; 2000.

(26) Hankey BF, Ries LA, Edwards BK. The Surveillance, Epidemiology, and End Results Program: a national resource. Cancer Epidemiology, Biomarkers & Prevention 1999;8:1117-21.

(27) Surveillance Research Program. About SEER. National Cancer Institute 2005; Available at http://seer.cancer.gov/about/. [Last accessed: 7-20-2005.]

(28) National Cancer Institute. Surveillance, Epidemiology, and End Results (SEER) Program (www.seer.cancer.gov) Public-Use Data (1973-2001), National Cancer Institute, DCCPS, Surveillance Research Program, Cancer Statistics Branch, released April 2004, based on the November 2003 submission. 2004; [Last accessed: 4-20-2004.]

(29) Cancer Statistics Branch Surveillance Program. SEER Extent of Disease -- 1988. Codes and coding instructions. Third edition. National Cancer Institute 1998; Available at http://seer.cancer.gov/publicdata/documentation.html. [Last accessed: 5-13-2005.]

(30) Asire A, Lyles B, Cicero BJ, Percy C, Cunningham J, Ries L, et al. The SEER program code manual. National Cancer Institute 1988; Available at http://seer.cancer.gov. [Last accessed: 5-13-2005.]

(31) Surveillance Research Program. Cancer registrar training. National Cancer Institute 2005; Available at http://seer.cancer.gov/training/. [Last accessed: 7-20-2005.]

(32) Warren JL, Klabunde CN, Schrag D, Bach PB, Riley GF. Overview of the SEER-Medicare data: content, research applications, and generalizability to the United States elderly population. Med Care 2002;40:IV-18.

(33) Du X, Freeman JL, Warren JL, Nattinger AB, Zhang D, Goodwin JS. Accuracy and completeness of Medicare claims data for surgical treatment of breast cancer. Med Care 2000;38:719-27.

(34) Cooper GS, Virnig B, Klabunde CN, Schussler N, Freeman J, Warren JL. Use of SEER-Medicare data for measuring cancer surgery. Med Care 2002;40:IV-8.

(35) Du X, Freeman JL, Goodwin JS. Information on radiation treatment in patients with breast cancer: the advantages of the linked Medicare and SEER data. J Clin Epidemiol 1999;52:463-70.

(36) Virnig BA, Warren JL, Cooper GS, Klabunde CN, Schussler N, Freeman J. Studying radiation therapy using SEER-Medicare-linked data. Med Care 2002;40:IV-49-54.

(37) Clark TG, Bradburn MJ, Love SB, Altman DG. Survival analysis part I: basic concepts and first analyses. Br J Cancer 2003;89:232-8.

(38) Kaplan E, Meier P. Nonparametric estimation from incomplete observations. J Am Stat Assoc 1958;53:457-81.

(39) Harrell FE, Jr. Regression modeling strategies with applications to survival analysis and logistic regression. Charlottesville, VA: University of Virginia; 2000. (Page 484-485).

(40) Therneau TM, Grambsch PM. Modeling survival data: extending the Cox model. New York, NY: Springer-Verlag; 2000. (Page 87-152).

(41) Overgaard M, Nielsen H, Overgaard J. Is the benefit of postmastectomy irradiation limited to patients with 4 or more positive nodes, as recommended in international consensus reports? A subgroup analysis of the DBCG 82 b & c randomized trials. Abstract 33. Radiother Oncol 2004;73, Suppl 1:S14-S15.

(42) van der Hage JA, Putter H, Bonnema J, Bartelink H, Therasse P, van de Velde CJ. Impact of locoregional treatment on the early-stage breast cancer patients: a retrospective analysis. Eur J Cancer 2003;39:2192-9.

(43) Woodward WA, Strom EA, Tucker SL, Katz A, McNeese MD, Perkins GH, et al. Locoregional recurrence after doxorubicin-based chemotherapy and postmastectomy: Implications for breast cancer patients with early-stage disease and predictors for recurrence after postmastectomy radiation. Int J Radiat Oncol Biol Phys 2003;57:336-44.

(44) Truong PT, Olivotto IA, Kader HA, Panades M, Speers CH, Berthelet E. Selecting breast cancer patients with T1-T2 tumors and one to three positive axillary nodes at high postmastectomy locoregional recurrence risk for adjuvant radiotherapy. Int J Radiat Oncol Biol Phys 2005;61:1337-47.

(45) Ellis IO, Schnitt SJ, Sastre-Garau X, Bussolati G, Tavassoli FA, Eusebi V, et al. Invasive breast carcinoma. In:Tavassoli FA, Devilee P, eds. Pathology and genetics of tumours of the breast and female genital organs. Lyon: IARC Press; 2003. (Page 13-59).

(46) Elston CW, Ellis IO. Pathological prognostic factors in breast cancer. I. The value of histological grade in breast cancer: experience from a large study with long-term follow-up. Histopathology 1991;19:403-10.

(47) Greene FL, Page DL, Fleming ID, Fritz AG, Balch CM, Haller DG, et al. AJCC Cancer Staging Handbook (6th Edition). New York: Springer Verlag; 2002. (Page 255-281).

(48) Sobin (Editor) LH, Wittekind (Editor) Ch. TNM Classification of Malignant Tumours, 6th edition. New-York: Wiley; 2002. (Page 131-141).
